# Mock-up pragmatic study on the impact performance of self-compacting concrete incorporating sea sand

**DOI:** 10.1038/s41598-024-75613-9

**Published:** 2024-10-21

**Authors:** B. M. Sindhurashmi, Gopinatha Nayak, N. D. Adesh, Sandhya Parasnath Dubey, Vidya Rao

**Affiliations:** 1 Department of Civil Engineering, Manipal Institute of Technology, Manipal Academy of Higher Education, Manipal 576104, Karnataka India; 2 Department of Information and Communication Technology, Manipal Institute of Technology, Manipal Academy of Higher Education, Manipal 576104, Karnataka India; 3 Department of Data Science and Computer Applications, Manipal Institute of Technology, Manipal Academy of Higher Education, Manipal, 576104 Karnataka India

**Keywords:** Self-compacting concrete, Sustainable concrete, Supplementary Cementitious Material(SCM), Class F Fly Ash (FA), Ground Granulated Blast Furnace Slag (GGBS), Friedel’s salt, Environmental sciences, Engineering, Materials science

## Abstract

Self-Compacting Concrete (SCC) allows for the use of non-desalted sea sand as a fine aggregate, but the durability of triple mix SCC with partial sea sand replacement remains unclear. To optimize binder and fine aggregate replacements, tests for consistency, setting times, soundness, compressive strength, and Ultrasonic Pulse Velocity were performed. Six SCC variations, incorporating 30$\%$ Class F Fly Ash (FA), 5$\%$ Ground Granulated Blast Furnace Slag (GGBS), and various fine aggregate combinations, were evaluated for their fresh, mechanical, microstructural, and durability properties. Results demonstrated that SCC with 50$\%$ sea sand and 50$\%$ manufactured sand achieved superior 90$^{th}$ day compressive strength. This improvement was attributed to accelerated cement setting and enhanced FA reactivity, leading to better hydration products. Microstructural analysis revealed more hydration products and fewer pores in specimens with 50$\%$ sea sand, due to the disconnected pore structure from Friedel’s salt formation. Chloride binding in concrete involves both chemical and physical mechanisms. Chemical binding is related to Friedel’s salt, while physical binding depends on Calcium-Silicate-Hydrate (C-S-H) content. Dense C-S-H formation from sea sand, confirmed by Scanning Electron Microscope (SEM) images, results in greater chloride binding. Additionally, aluminum oxide in FA and GGBS enhances chemical binding by forming Friedel’s salt.

## Introduction

The progress of a country is precisely relying on the available infrastructure. Subsequently, advancements in the improvement of the infrastructure have resulted in the increased demand for construction materials like river sand and cement. This increased demand for building materials leads to many adverse environmental effects, depletion of river sand, and also resulted in the increased price of building materials^1^. In response, many governments have recently imposed bans on unauthorized river sand mining to tackle these issues^2^. This has resulted in shortages of river sand for making concrete, creating havoc. Additionally, the frantic surge in industrialization and urbanization has significantly increased the production of industrial by-products.

According to the World Bank Group’s 2018 assessments^2^, global industrial waste generation is predicted to reach 2.59 billion tons by 2030, with expected growth to 3.4 billion tons by 2050. The inappropriate disposal of these industrial by-products presents substantial risks to both human health and the environment, thereby necessitating the development of effective disposal methods, which undoubtedly involve considerable expense and challenge. Infrastructure advancements have led to a higher need for construction materials such as river sand and cement^1^. This has prompted an increasing interest for utilizing diverse industrial by-products in concrete and incorporating alternative fine aggregates.

As per the Intergovernmental Panel on Climate Change (IPCC), founded by the World Meteorological Organization, the increase in socio-economic changes over the past 40 years has resulted in the alteration of the earth’s environment. Cement production alone produces for about $8\%$ of the total greenhouse gas emissions ($CO_{2}$)^3^. In this context, concrete industries lead to environmental changes. Additionally, the use of alternative materials in construction will lead to a reduction in $\hbox {CO}_2$ emissions. Supplementary Cementitious Materials (SCMs) are by-products obtained from either industry or agriculture. Examples include Rice Husk Ash (RHA), Fly Ash (FA), Silica Fume (SF), Ground Granulated Blast Furnace Slag (GGBS), and Metakaolin. These materials can be effectively used in concrete as alternatives to disposal^4^. Subsequently, there will be the production of Calcium-Silicate-Hydrate (C-S-H) and Calcium-Hydroxide $Ca(OH_{2})$ during the hydration process of Portland Cement. Calcium Silicate Hydrate (C-S-H) gives strength to concrete, but Calcium Hydroxide is responsible to reduce the strength of concrete. In this context amorphous silica present in SCMs react with Calcium Hydroxide and convert into C-S-H when SCMs are added to Portland Cement (PC). Thus enhancing concrete properties due to the pozzolanic effect and filling effect. SCMs reduces permeability and improves the durability of concrete^4–6^.

The region where rivers join the sea is called an estuary. Sand is a significant economic resource in demand for a wide range of uses. Sedimentation involving silt transportation which regularly carries nutrients from upstream to downstream of a river stream where it joins the sea in huge quantity. One of the large portions of these sediments is sand. This sedimentation at an estuary decreases the storage capacity by increasing the bed level. This will greatly affect the biotic life, increases flood plains, the mouth of the estuaries displacement^7^. This being the reason, sand collected at estuaries during post and pre-monsoon time can be utilized for infrastructure or housing needs due to the lesser salinity^8^. Furthermore, the mining of the sea sand from this place should be lesser than the quantity of the sand yielded to reduce over-exploitation of the sea sand and to achieve sustainability. The use of dredged marine sand, with a geological origin similar to river sand, has been a long-standing practice. While desalted sand is extensively used in countries like Japan for concrete production, it adds extra costs and impedes construction efficiency. Replacing depleting river sand with raw marine sand can effectively reduce construction costs, especially in coastal areas where transportation costs are reduced^9^. The incorporation of sea sand and sea water in concrete production yielded a compressive strength of 50.90 MPa, while plain concrete demonstrated a slightly lower compressive strength of 49.34 MPa^10^. The results imply that the presence of sea sand and sea water does not have a substantial impact on the concrete’s strength. Aforementioned results reinforces the feasibility of employing sea sand in the production of Self-Compacting Concrete (SCC). Concrete that requires minimal vibration or compaction was practised in Europe in the early 1970s but was not adopted in Japan until the late 1980s. In 1989, Okamura and Ozawa introduced SCC in Japan^11^. This innovative concrete type can self-compact under its own weight, even in the presence of intricate reinforcements, eliminating the need for mechanical vibrators^12^.

Although researchers have separately investigated FA and GGBS as cement substitutes, there is limited research on the combined use of these alternatives to Ordinary Portland Cement (OPC) in SCC production with sea sand. Based on insights from existing literature, there is a scarcity of durability studies investigating the implications of utilizing a blend of sea sand and Manufactured sand (M sand) in triple mix SCC production that incorporates sea sand. There is a need for a comprehensive analysis of sea sand’s impact on SCC properties, alongside the identification of optimal replacements for both Supplementary Cementitious Materials (SCMs) and sea sand itself. While major previous studies^9,13–19^ have explored the utilization of sea sand in concrete to the best of our knowledge. However, the results are mixed. Vitalii Kryzhanvskyi et al.^18^ found that incorporating sea sand increased the slump flow of SCC, while another study^17^ reported reduced workability in fresh concrete. Additionally, knowledge on improving the durability of SCC incorporating sea sand through chloride binding is limited in previous studies. Furthermore, the durability properties of SCC prepared with partial sea sand replacement, alongside manufactured and river sand, remain unclear in triple mix SCC production, which is crucial for practical engineering applications. This has inspired the development of a new type of SCC to enhance environmental sustainability, durability, and reduce construction costs. The developed triple mix SCC aims to achieve three objectives: (1) To investigate the optimum replacement of sea sand and SCMs through mortar studies; (2) To study the fresh properties of the SCC mixes with the ternary binder and mechanical properties of hardened SCC mixes with the ternary binder by conducting workability and strength tests; (3) To evaluate the durability properties of six varieties of the developed SCC mixes and compare the results. To achieve the aforementioned goals, it is necessary to develop triple mix M40 grade SCC incorporating sea sand and a ternary binder that adheres to EFNARC 2005^12^ and IS 10262:2019^20^ standards. The contributions of the manuscript are as follows:

1. The investigation of optimal replacements for SCMs and sea sand through mortar studies aims to utilize alternative materials in construction. This approach seeks to reduce $\hbox {CO}_2$ emissions and address the declining availability of river sand. Sea sand is incorporated as a fine aggregate in SCC production to achieve these goals.
2. Development of six innovative triple mix SCC mixes are designed to evaluate the fresh, hardened, and micro-structural properties of SCC incorporating sea sand and other variations of fine aggregates.
3. The construction industry generally requires 6 to 10 tonnes of gravel for every tonne of cement used. With global cement production reaching 4.1 billion tonnes in 2017 across 150 countries, the total demand for concrete production is estimated to be around 28.7 to 32.8 billion tonnes^21,22^. To counter this problem, the evaluation of the durability of the developed triple mix SCC incorporating sea sand was conducted through various durability tests. Lower porosity, facilitated by Friedel’s salt formation, contributed to matrix densification, observed in water absorption, sorptivity, Rapid Chloride Permeability Tests (RCPT), Scanning Electron Microscope (SEM) images, Energy Dispersive Spectroscopy (EDS) analysis, and X-Ray Diffraction (XRD) analysis. Additionally, the SCC mix with 50$\%$ sea sand performed well in sulphuric acid and sulphate attack tests. Enhanced durability properties of the triple mix SCC incorporating 50$\%$ sea sand and 50$\%$ manufactured sand suggest that substituting river sand with sea sand could help decrease the reliance on river sand.

The rest of the manuscript is organized as follows: Section II covers related work, Section III describes the methodology, Section IV details the materials, and Section V outlines the testing program. In Section VI, results and discussions are presented. Conclusions are provided in Section VII, and future perspectives are outlined in Section VIII.

## Related work

Significant research has been conducted on the development of SCC^23–26^. However, incorporating SCMs into SCC does not address the issue of diminishing river sand. Recently, a limited amount of research has focused on substituting fine aggregate with sea sand. Using 40$\%$ Recycled Concrete Aggregate (RCA) as a replacement for coarse aggregate, along with the addition of 3$\%$ Silica Fume (SF) and 20$\%$ Fly Ash (FA) to Recycled Aggregate-Seawater-Sea sand Concrete (RASSC), yielded a significant compressive strength of 47.7 MPa, showcasing the effectiveness of the dual mineral admixture in improving concrete strength^27^. Concrete made with natural sea sand demonstrated better mechanical characteristics in comparison to concrete produced using desalinated sea sand, irrespective of curing period, utilization of Fly Ash (FA) or Blast Furnace Slag (BFS) as substitutes^28^. The study discovered that the existence of chloride ions derived from Dredged Marine Sand (DMS) led to a reduction in the carbonation of DMS concretes by 20$\%$ to 50$\%$. This increased resistance to carbonation serves to mitigate the risk of steel reinforcement deterioration^29^. According to multiple research findings, salt demonstrates limited permeability and porosity, mainly due to the absence of significant open voids within the substance^30^.

Tjaronge et al.^31^ conducted a study to assess the impact of sea sand and seawater on the production of SCC. Their findings revealed a satisfactory compressive strength of approximately 42.5 MPa after 28 days^31^. The study observed that the development of C-S-H was not hindered by the reduced production of Calcium Hydroxide $(\text {Ca(OH)}_2 / \text {CH})$, as there was an increased production of ettringite and Fridel’s salt from the 1$^{st}$ day to the 28$^{th}$ day. Also, the researchers empirically examined concrete performance characteristics by considering the inclusion of sea sand and seawater using two distinct water-to-binder ratios: 0.38 (group A) and 0.28 (group B)^32^. Reference concrete (No. 0) was produced by mixing river sand and fresh water, while sea sand concrete (No. 1) was produced by mixing sea sand and fresh water. Seawater and sea sand concrete (No. 2) were produced by mixing seawater and sea sand. The study used Ordinary Portland Cement (OPC), Silica Fume (SF)^33–35^, and Fly Ash (FA) as binding materials. Compared to reference concrete, the compressive strength of concrete mixes containing seawater and sea sand at the specified water ratios was higher^32^. The study also noted the acceleration of the hydration reaction due to the presence of highly soluble salts found in seawater and sea sand^32,36^. Previous research shows that replacing fine aggregate with up to 100$\%$ sea sand improves the split tensile and compressive strengths of concrete due to the pore refinement achieved by the finer particles in the sea sand^37^.

**Figure 1:**
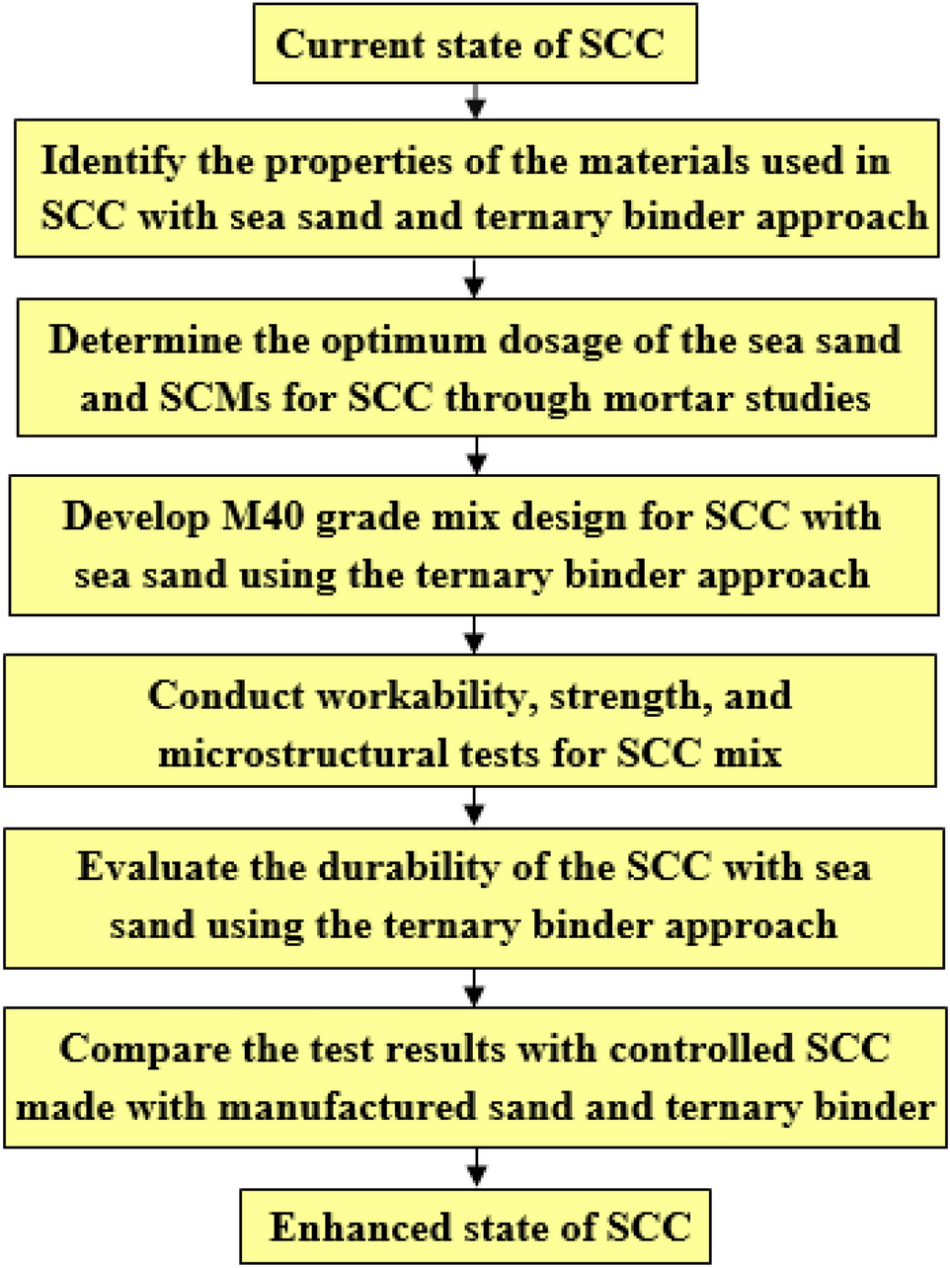
Methodology.

The studies revealed that incorporating sea sand and seawater does not impact the concrete’s strength^32,38^. Also, the surface texture of sea sand played a role in enhancing the connection between material particles and the hydrated gel^39^.

**Figure 2:**
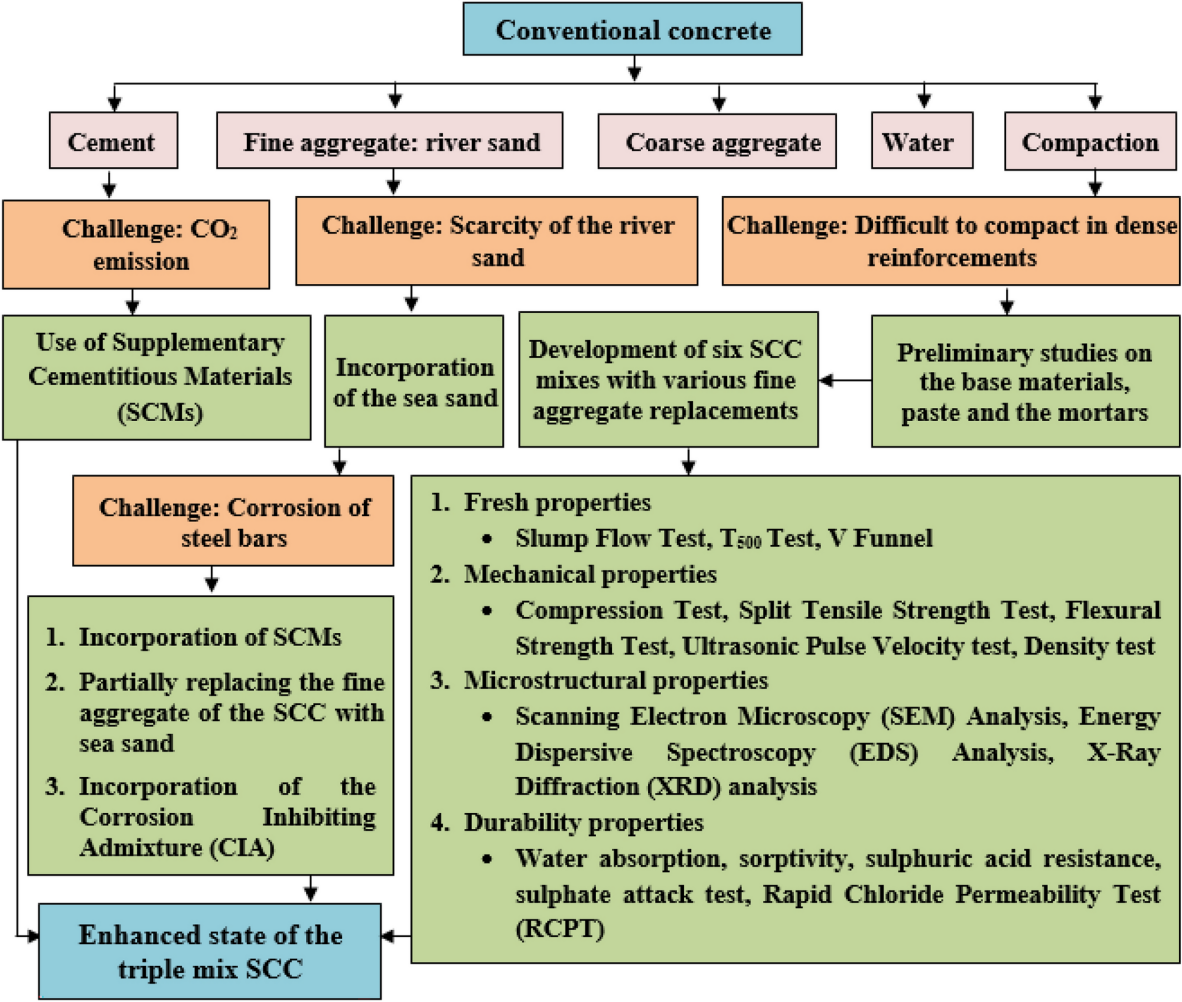
Flowchart for developing durable triple mix SCC.

An extensive literature survey on the utilization of sea sand as a replacement for river sand indicates a lack of solid comprehensive investigation into the durability properties and chloride binding mechanisms of triple mix SCC, which incorporates various fine aggregate variations alongside sea sand.

## Methodology

The proposed methodology aims to address the objectives of the pragmatic study by designing the SCC mix using sea sand and ternary binder approach, thereby preserving the natural sand of the river banks. Meanwhile, usage of FA and GGBS will help in reducing embodied energy and thus reduces $CO_{2}$ emission. The proposed methodology is as shown in Figure 1. First step in the methodology is to identify the properties of the materials used in SCC with sea sand and ternary binder. Second step is to determine the optimal dosage of sea sand and SCMs for SCC through mortar studies. Third step is to develop the M40 grade mix design for SCC with sea sand using a ternary binder. Fourth step is to conduct a workability and strength test for the SCC mix. Fifth step is to evaluate the durability of the SCC with sea sand using a ternary binder. A comparison of the test results with controlled SCC made with manufactured sand and ternary binder will be conducted in the sixth step. In this manner, the methodology ensures the achievement of objectives for triple mix SCC, enhancing the performance of SCC with sea sand. Additionaly, Figure 2 illustrates the flowchart for developing durable triple mix SCC.

**Table 1:** Distinctive characteristics of the binding materials.

Physical properties	Fly ash	GGBS	OPC
Specific surface area ($m^2$/kg)	-	-	346
Colour	Dark grey	Off white	Grey
Water requirement	Reduces	Depends on the fineness	-
Shape	Spherical	Angular	Crystallized structure
Cement Replacement ($\%$)^20^	15-30	25-50	NA
Size	1-100 $\mu$m	-	-
Specific gravity	2.12	2.85	3.15
Particle size $d_{10}$ ($\mu$m)	2.94	2.51	2.66
Particle size $d_{50}$ ($\mu$m)	17.93	15.64	17.42
Particle size $d_{90}$ ($\mu$m)	100.50	44.29	53.80
Maximum residue retained on 45 micron sieve $\%$	19.50	-	-
Moisture content $\%$	0.13	0.10	-

**Table 2:** Chemical properties of the binding materials.

Chemical properties ($\%$ by mass)	Fly ash	GGBS	OPC
Silicon dioxide ($Si O_{2}$)	57.31	-	-
Silicon dioxide ($Si O_{2}$) $+$ Alumina ($Al_{2}O_{3}$) $+$ Iron Oxide ($Fe_{2}O_{3}$)	92.75	-	-
CaO $$+$$ MgO $$+$$$$Si O_{2}$$	-	76.03	-
(CaO $+$ MgO) / $Si O_{2}$	-	1.30	-
CaO / $Si O_{2}$	-	1.07	-
Sulphide Sulphur	-	0.50	-
Sulphite Sulphur	-	0.38	-
Glass content	-	91	-
Manganese content	-	0.12	-
Insoluble residue	-	0.49	2.16
Tricalcium Aluminate ($\hbox {C}_{{3}}$A)	-	-	5.79
Alumina $Al_{2}O_{3}$	-	-	1.02 (Alumina modulus)
*CaO*	-	-	0.90 (Lime Saturation Factor )
Magnesium Oxide *MgO*	0.89	7.73	1.09 (Magnesia)
Total sulphur as Sulphur Trioxide $SO_{3}$	0.38		2.88 (Sulphuric Anhydride)
Total Chlorides	0.01	0.01	0.04
Titanium ($TiO_{2}$) mg/kg	956	-	-
Phoshorous ($P_{2}O_{5}$) mg/kg	5188	-	-
Loss On Ignition (*LOI*)	3.09	0.26	3.63

## Materials

The distinctive characteristics of OPC 53 grade, GGBS, Class F Fly Ash (FA), fine aggregates, coarse aggregate, water, and chemical admixture were studied to formulate the triple-mix SCC.

**Figure 3:**
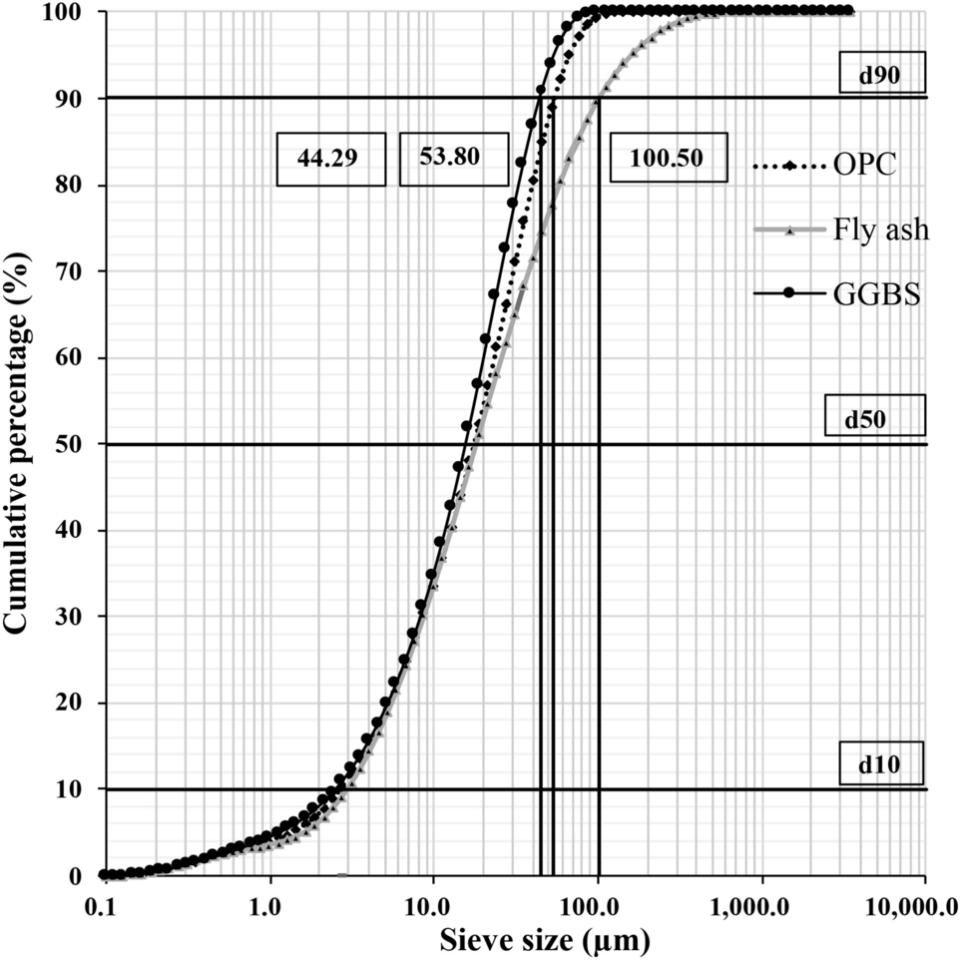
Particle size distribution of the binders.
  The choice of OPC 53 grade was made due to the requirement for higher strength as specified in IS 269^40^. To improve the durability of sea sand concrete, chloride binding is crucial. Adding alumina-rich SCMs like FA and GGBS influences the Ca/Al ratio in the cement system. An optimal Ca/Al ratio enhances chloride binding^41^. As per the study^42^, including GGBS and FA led to the generation of additional hydration products and denser structures, and improved the Interfacial Transition Zone (ITZ). As per the investigation^43^, concrete with GGBS and FA preserved its compressive strength in the marine environment, based on the findings acquired from the on-site samples. The incorporation of blast furnace slag powder or fly ash into sea-water concrete, as highlighted in studies^41,44^, significantly enhanced the corrosion resistance of steel bars and reduces material penetrability. These observations from the previous studies strengthen the selection of binders in the proposed study.
  From these inferences, OPC was replaced with 10$\%$ to 50$\%$ fly ash, and GGBS was replaced with 5$\%$ to 15$\%$ in the development of the triple mix mortar cubes.
- **B]Aggregates**
  A combination of untreated sea sand (Zone 3) and manufactured sand (Zone 3) was selected as fine aggregates. The sea sand was sourced from Padukere Beach, Udupi, Karnataka, India. Coarse aggregates, ranging in size from 10 mm down, were used for the pragmatic study. Additionally, river sand (Zone 1) was used as a fine aggregate for comparative analysis. The particle size distribution of the aggregates is as shown in Figure 4.

- **A]Binders**
  The distinctive characteristics of the binders are presented in Table 1 and Table 2. The particle size distributions of the binders are as shown in Figure 3.

**Figure 4:**
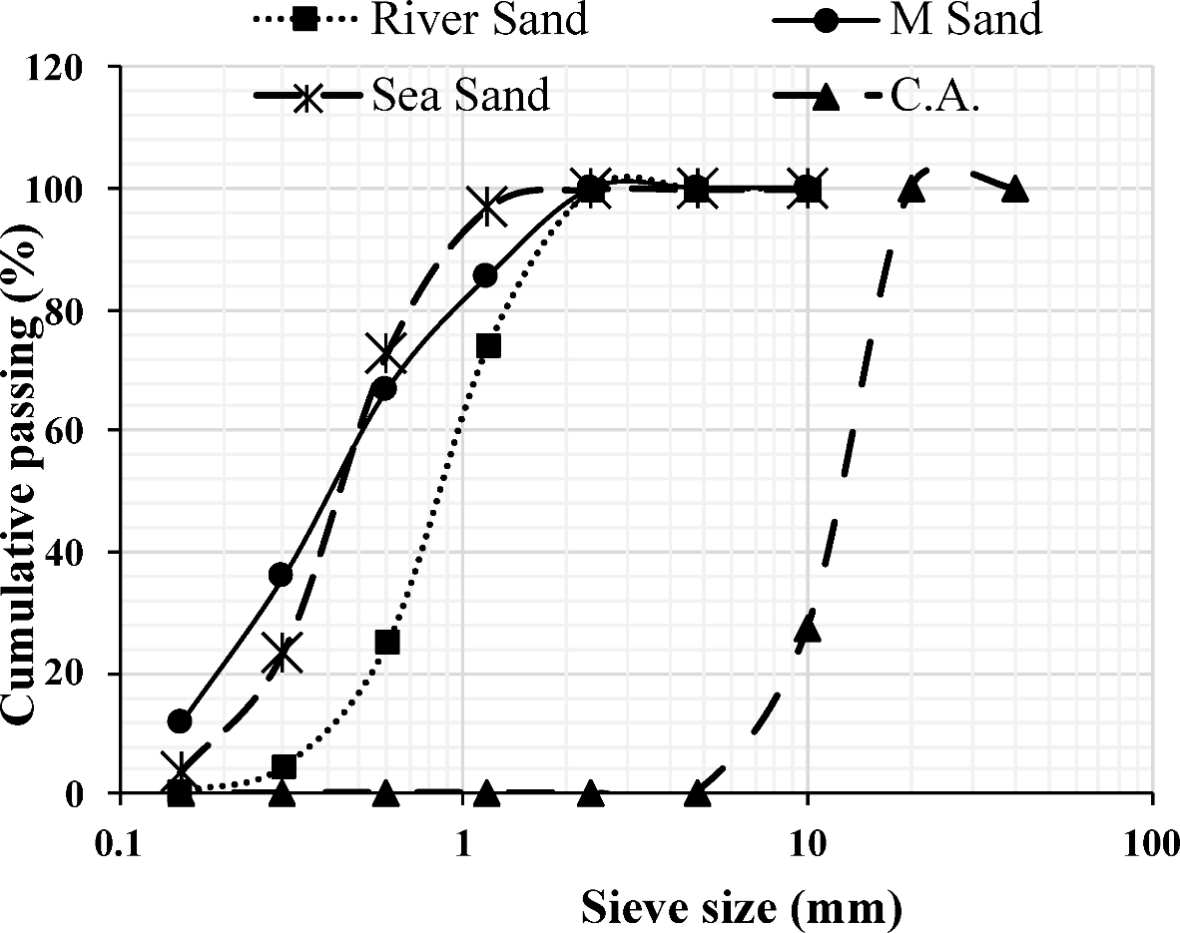
Particle size distribution of the aggregates.
  IS 456 2000 specifies that the acid-soluble chloride content of reinforced concrete or plain concrete with metal should be lower than $0.6\hbox { kg/m}^{3}$^11,45^. Allowable ranges for the characteristics of sea sand as per JGJ 206-2010^9,46^ are as shown in Table 3.

**Table 3:** Permissible limits of the properties of the sea sand^9,46^.

Properties	Permissible Limits
Sediment Content	$$\le$$$$1.0\%$$
Sea shell content	$$\le$$$$3.0\%$$
$$\hbox {Cl}^-$$	$$\le$$$$0.03\%$$
$$SO_{3}$$ / $$SO_{4}^{2-}$$	$$\le$$$$1.0\%$$
Ruggedness	$$\le$$$$8.0\%$$
- **Microstructure assessment of the fine aggregates**
  A material’s microstructure will be defined by a microscopic enlargement. The microstructure is responsible for any material’s physical properties. Electron microscopic methods will be used to obtain high-resolution details on the mineralogical microstructure^47^.
  - **Scanning Electron Microscopy (SEM) analysis**
    SEM aimed to comprehend the texture of various fine aggregates, as depicted in Figure 5. The SEM analysis from Figure 5 (a) and Figure 5 (d) revealed the irregular texture of the sea sand, aligning with observations from the study^39^, which emphasized the irregular nature of sea sand texture. As reported in the study^39^, the irregular texture of sea sand strengthened the connection between material particles and the hydrated gel, leading to enhanced packing.
    From the SEM analysis illustrated in Figure 5 (b), manufactured sand was found to be more angular in shape, while river sand particles exhibited rounded or sub-angular shapes, as shown in Figure 5 (c). Furthermore, the sea sand particles displayed a variety of shapes, including angular, sub-angular, and rounded, resulting from the erosion caused by ocean currents and waves on rocks and minerals, as illustrated in Figures 5 (a) and 5 (d).
  - **X-Ray Diffraction (XRD) analysis**
    The XRD patterns reveal that sea sand, river sand, and manufactured sand exhibit comparable mineral compositions, primarily comprising quartz and feldspar, as shown in Figure 6. Similar results were found in study^29^.
- **C]Water**
  The concrete was mixed and cured using potable water with a pH of 7.01, measured using a pH meter.
- **D]Chemical admixture**
  A polycarboxylic ether-based super-plasticizer (SP) with a relative density of 1.10 ± 0.02 at 25$^{\circ }$ C was used as a chemical admixture to enhance the fresh characteristics of the SCC.

**Figure 5:**
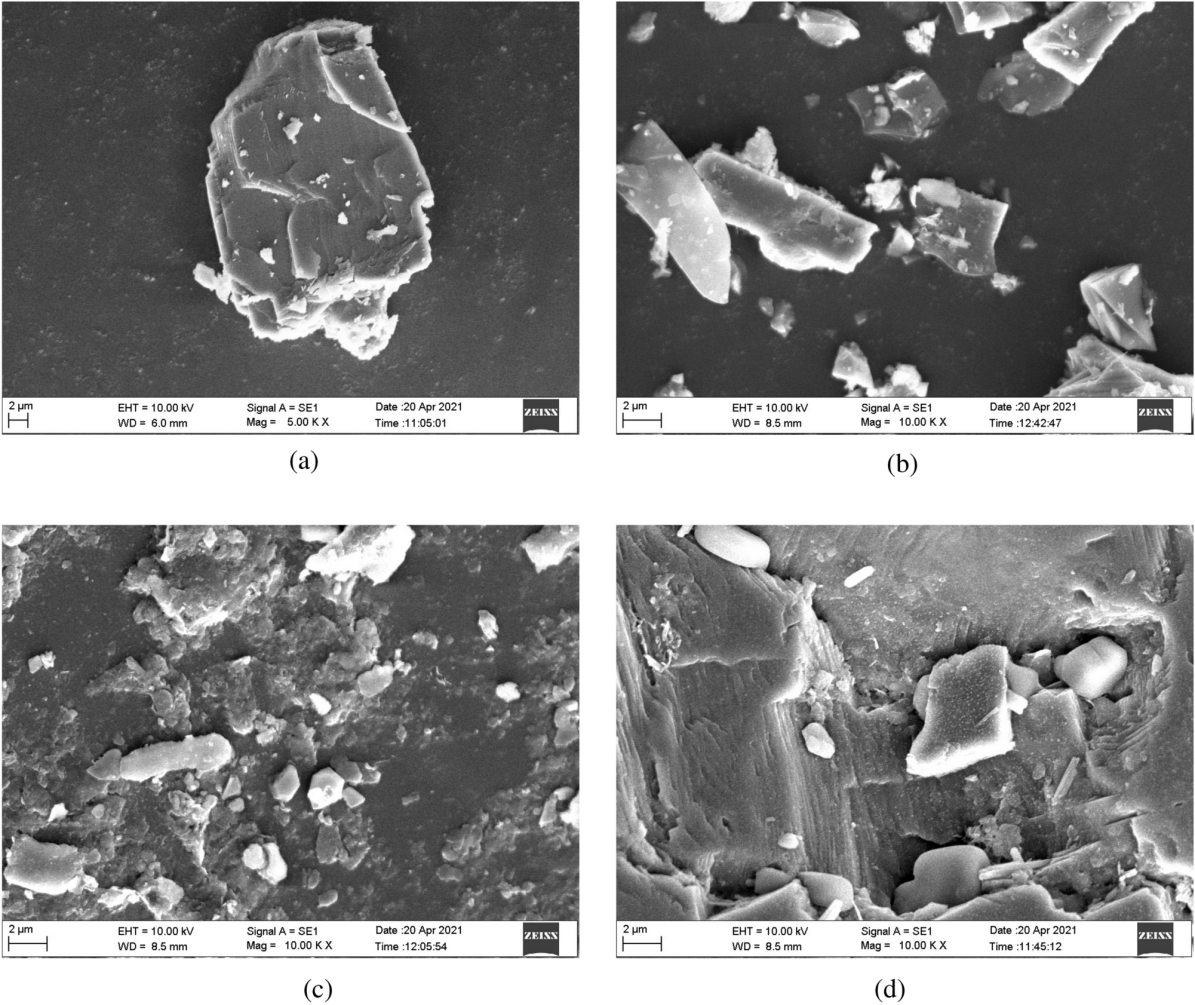
SEM images of the fine aggregates.

**Figure 6:**
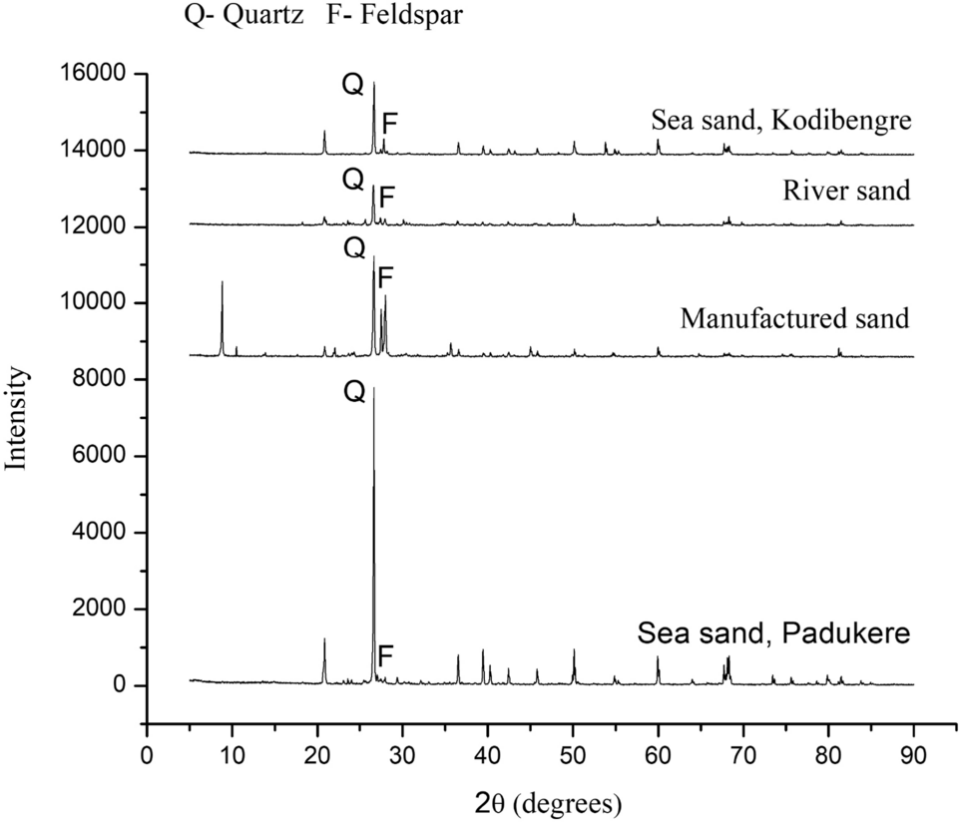
XRD spectra of fine aggregates.

**Table 4:** Mix proportions and the designations of the mortar mixes with varying binder and sea sand content.

Mix designation	Sample Type	p $\%$	Cement($\%$)	Fly ash ($\%$)	GGBS ($\%$)	M sand ($\%$)	sea sand ($\%$)	river sand ($\%$)
NM	Control	32	100	-	-	100	-	-
TMF10G5	FA Var.	33	85	10	5	100	-	-
TMF20G5	FA Var.	34	75	20	5	100	-	-
TMF30G5/ TMF30G5M100	FA Var.	35.6	65	30	5	100	-	-
TMF40G5	FA Var.	38.3	55	40	5	100	-	-
TMF50G5	FA Var.	39	45	50	5	100	-	-
TMF30G10	GGBS Var.	35.6	60	30	10	100	-	-
TMF30G15	GGBS Var.	36	55	30	15	100	-	-
TMF30G5R100	Control	35.6	65	30	5	-	-	100
TMF30G5S100	Control	35.6	65	30	5	-	100	-
TMF30G5M100	Control	35.6	65	30	5	100		
TMF30G5-S10	Sea sand Var.	35.6	65	30	5	90	10	-
TMF30G5-S20	Sea sand Var.	35.6	65	30	5	80	20	-
TMF30G5-S30	Sea sand Var.	35.6	65	30	5	70	30	-
TMF30G5-S40	Sea sand Var.	35.6	65	30	5	60	40	-
TMF30G5-S50	Sea sand Var.	35.6	65	30	5	50	50	-
TMF30G5-S60	Sea sand Var.	35.6	65	30	5	40	60	-
TMF30G5-S70	Sea sand Var.	35.6	65	30	5	30	70	-
TMF30G5-S80	Sea sand Var.	35.6	65	30	5	20	80	-
TMF30G5-S90	Sea sand Var.	35.6	65	30	5	10	90	-

Normal Mix - NM, Triple Mix - TM, Fly Ash- F, GGBS- G, Consistency- p.

## Testing Program

Various experimental methods have been employed to study the ternary paste and mortar mixes. Additionally, the fresh properties, mechanical properties, microstructural properties, and durability properties of the triple-mix SCC were evaluated. The mix proportions and the designations of the mortar mixes with varying binder and sea sand content are shown in Table 4.

- **A]Mix proportions for M40-grade triple mix SCC incorporating sea sand**
  The primary objective of the study was to develop sustainable M40-grade SCC using the volume batching method, following IS 10262:2019^20^ and EFNARC 2005^12^ guidelines. The main reason for choosing this grade is that replacing depleting river sand with raw marine sand can effectively reduce construction costs, especially in coastal areas where transportation costs are lower^9^. In this context, the minimum grade of concrete for extreme exposure (surfaces of structures in the tidal zone and those in direct contact with harsh chemicals), as per IS 456, is M40^11^. The target compressive strength for the control mix TMSM100 at 28 days was aimed at 48.25 MPa, in accordance with IS 456 guidelines^11^.
- **B]Tests conducted on the fresh properties of SCC**
  SSC mixes were tested for workability using various tests, including the slump flow test, $T_{500}$ Test, V funnel test, following the EFNARC 2005 guidelines. The classification employed in the SCC specification is presented in Table 5. Where, $\hbox {SF}_{{1}}$ to $\hbox {SF}_{{3}}$- Consistence classes expressed by slump-flow.
- **C]Hardened concrete tests for studying mechanical properties of triple-mix SCC**
  The mechanical behavior of the hardened SCC mixes was determined through compression test^48^, split tensile strength test^49^, and flexural strength test. Specimens subjected to Ultrasonic Pulse Velocity (UPV), compression strength, split tensile strength, and flexural strength tests.
  - **Compressive strength**
    Compressive strength was calculated from 100 mm SCC cubes cured for 7, 28, 56, and 90 days.
  - **Splitting strength**
    The cylindrical samples with dimensions of 150 x 300 mm were used for the test.
  - **Flexural strength of concrete**
    The test was conducted on beams measuring 100 x 100 x 500 mm in size. The Figure 14 displays flexural strength results of various triple-mix SCC samples, where the specimens fractured within the central one-third of the span length.
- **D]Evaluation of the quality of SCC through a non-destructive approach**
  - **Ultrasonic Pulse Velocity (UPV) test**
    The UPV test is a non-destructive approach used to assess concrete quality. Higher velocities measured by the UPV test indicate superior density, uniformity, and homogeneity^50^. Concrete quality grading based on UPV test results is presented in Table 6.
- **E]Microstructural analysis**
  The sample will be scanned with a focused beam of electrons to obtain images. The interaction between the electrons and the atoms in the sample will produce signals that provide information on the sample’s morphology, topography, and crystallography. Additionally, Energy Dispersive Spectroscopy (EDS) and X-Ray Diffraction (XRD) will provide information on the minerals present in the samples^47^. Furthermore, Scanning Electron Microscopy (SEM) analysis provides information on the material’s topography.
  - **Scanning Electron Microscope (SEM) analysis**
    The internal behaviour of various triple mix SCC specimens aged for 90 days under water curing was analyzed using SEM.
  - **Energy Dispersive Spectroscopy (EDS) analysis**
    The mineralogical composition of the sample can be analyzed with EDS using high magnification by detecting the X Rays emitted by the sample^47^.
  - **X-Ray Diffraction (XRD) analysis of SCC mixes**
    The powdered sample will be kept on the XPERT- 3 diffractometer. Radiation with angle scanning will be conducted. Scattering of X rays due to atoms will indicate the lattice spacing of the elements present in the sample. Different angle intensities will be correlated with a database of various minerals and compounds^47^.
- **F]Durability characteristics**
  The Durability of concrete is firmly related to the permeability of the outer most layer of concrete, which should restrict the entry of the materials like $CO_{2}$, chloride, sulphates, water, alkalis, acids which is capable of destroying the concrete. The durability depends on the type of the material, quality of the material, compaction, finishing, careful concrete placement^12,33^.
  - **Water absorption and density of SCC**
    The water absorption test was performed on the hardened concrete cubes of 100 mm size as per BS 1881: Part 122, 1983, and ASTM C642-21^33,45,52^.
  - **Sorptivity**
    This method measures the water absorption rate of both the surface and interior of concrete^53^. The test is conducted as per ASTM C1585-20^53^ on cylindrical samples with a 100 mm diameter and 50 mm height.
  - **Sulphuric acid resistance of hardened SCC mixes**
    Cube specimens measuring 100 x 100 x 100 mm will be cast and stripped once fully hardened. They are then immersed in water for 28 days. Cubes will be dipped into $5\%$ sulphuric acid completely for 28, 56, and 84 days. The loss of mass of the sample will be due to the acid attack^33^. Hardened SCC specimens exposed to sulphuric acid for 84 days are as shown in Figure 22. Interpretation of the results were made as per ASTM C1898-20^54^.
  - **Sulphate attack test on hardened SCC mixes**
    Sulphate resistance is vital for the durability of marine concrete. When sulphate ions infiltrate the concrete, they often lead to the formation of expansive products such as ettringite, gypsum, and other salt crystals, which can severely damage the concrete^55^. Resistance to sulphate attack can be calculated by submerging a 100 mm concrete cube in a water tank having $5\%$ sodium sulphate for 28, 56 and 84 days. Weakening of cube specimens will be examined by the loss of compressive strength^33^. Also, interpretation of the results were made as per ASTM C267-20^56^. Hardened SCC specimens exposed to sulphate attack for 84 days are as shown in Figure 23.
  - **Rapid Chloride Permeability Test (RCPT)**
    The test is conducted as per ASTM C1202-22^57^ on cylindrical samples with a 100 mm diameter and 50 mm height. Cylindrical samples will be moist cured for 28 days.

**Table 5:** Slump-flow classes^12^.

Class	Slump-flow in mm
SF$_{1}$	550 to 650
SF$_{2}$	660 to 750
SF$_{3}$	760 to 850

**Table 6:** Concrete quality grading based on the UPV test^51^.

UPV results in $\text {km/s}$	Concrete quality grading
Above 4.40	Excellent quality
3.75 to 4.40	Good quality
3.00 to 3.75	Doubtful quality
Below 3.00	Poor quality

## Results and discussions

**Figure 7:**
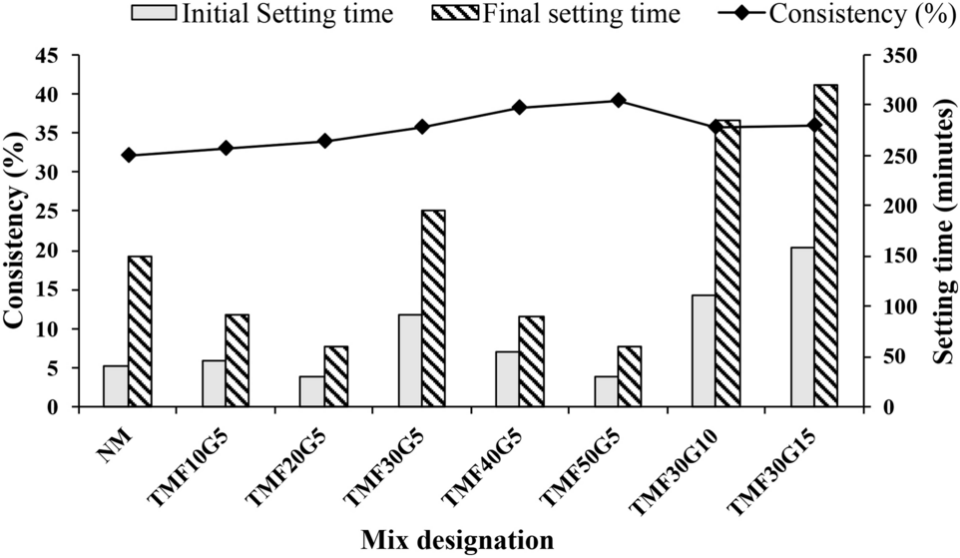
Consistency and setting times (initial and final) of triple mix binders.
- **B]** **Soundness test**
  The soundness values of the triple mix cementitious blends are depicted in Figure 8, and the values were within the limit of 10 mm.

- **A]** **Consistency and setting time**
  The consistency and setting times of the binder mixes are shown in Figure 7. The OPC (NM) mix showed a consistency of 32$\%$. With increasing fly ash and GGBS content in the triple-mix cement blend, consistency values gradually increased, attributed to the greater surface area of fly ash and GGBS, as illustrated in Figure 7. Additionally, the setting time results obtained for all the mixes were within the limits provided by the IS 12269, 2013^58^.

**Figure 8:**
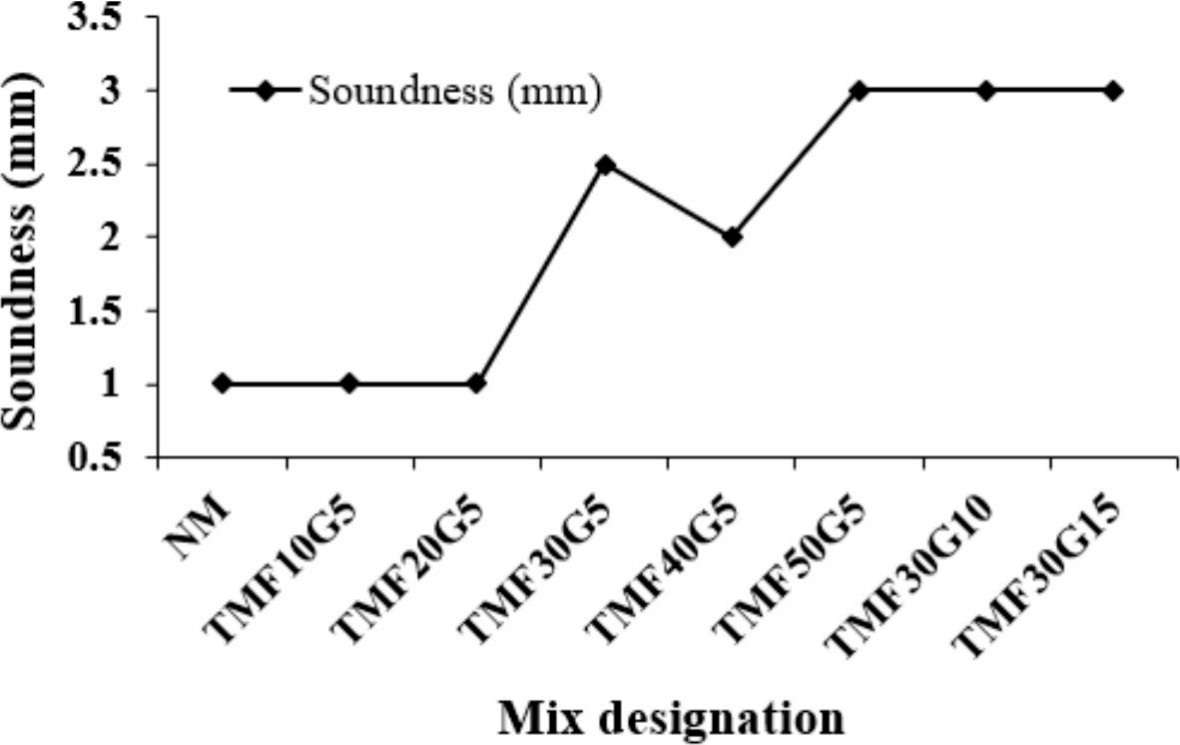
Soundness values of the triple mix binders.
- **C]** **Study on mortar cubes to evaluate the optimal dosage of supplementary cementitious materials (SCMs) and sea sand**
  In the proposed triple mix SCC, OPC 53 grade cement serves as the primary binder, while fly ash (30$\%$) functions as the secondary binder. Additionally, GGBS (5$\%$) acts as the ternary binder in the triple mix SCC.
- **D]** **Compression strength test results of mortar cubes to determine the optimum replacement of SCMs and sea sand**
  - **Compression strength test results on mortar cubes with varying binder content**
    Compression strength test values for the mortar cubes with varying binder content are presented in Figure 9 (a). Since it was the sustainable triple mix, the SCC mix designation TMF30G5 with $28^{th}$ day compressive strength of 19.80 N/$mm^2$ was considered for the pragmatic study. The result aligns with study^23^, indicating that the combination of FA with Portland Pozzolana Cement (PPC) effectively produces high-strength and high-performance SCC. Substituting 30$\%$ of PPC with FA resulted in a strength of approximately 100 MPa on the $56^{th}$ day. Among the variations in GGBS content at 5$\%$, 10$\%$, and 15$\%$, in combination with 30$\%$ FA, it was found that the mix containing 5$\%$ GGBS exhibited the highest compressive strength of 19.80 N/$mm^2$ on the $28^{th}$ day. However, for the proposed study, it was found that a mix of 30$\%$ FA and 5$\%$ GGBS resulted in the highest strength on the $28^{th}$ day. Additionally, as per IS 10262:2019, clause 6.2.6, the recommended total fly ash content is 15-30$\%$ of the total cementitious material^20^, thus justifying further investigation in SCC studies.

**Figure 9:**
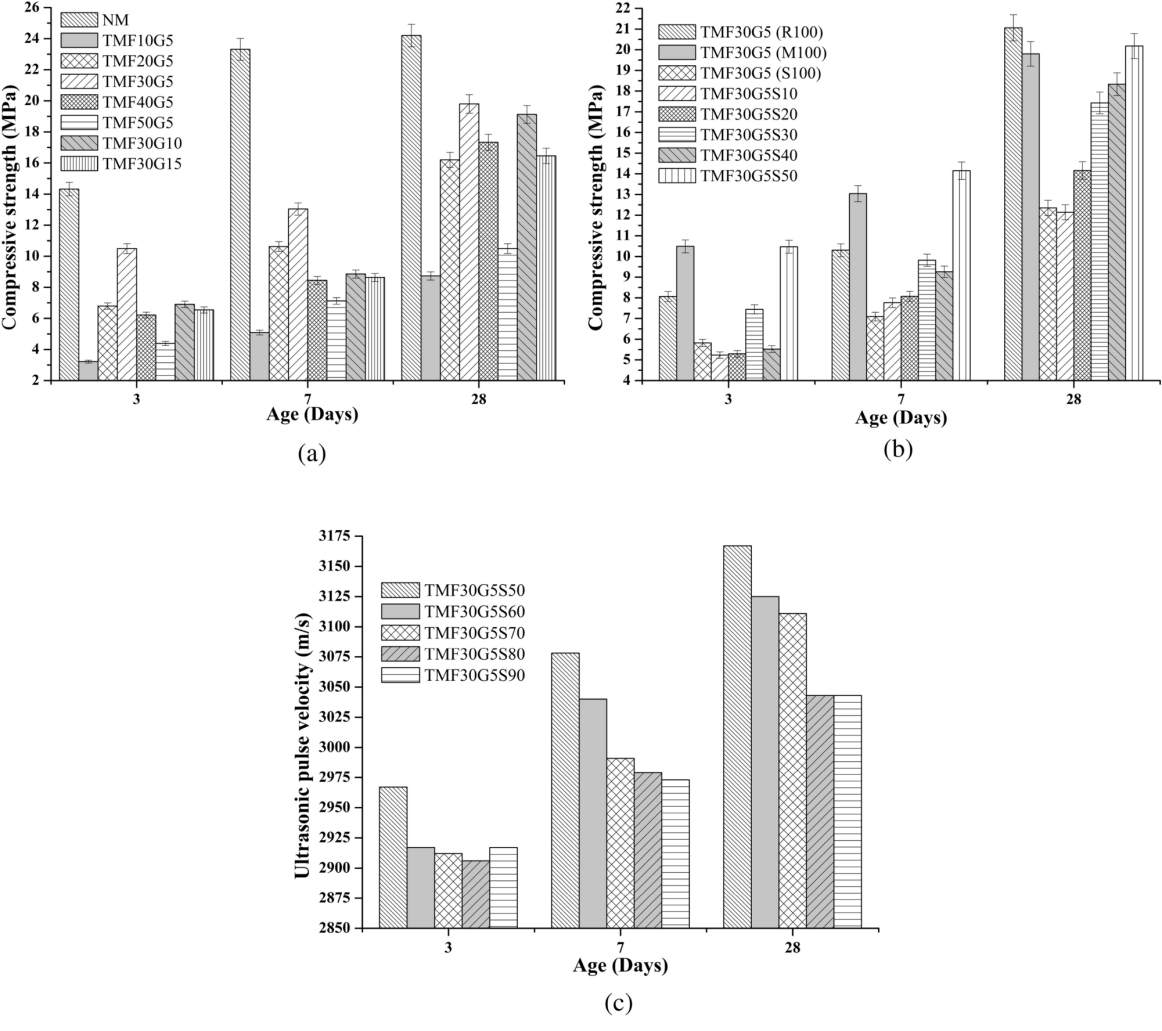
Compressive strength and UPV values of various mortar cubes to determine the optimal binder content and sea sand replacement.
  - **Compression strength test results and Ultrasonic Pulse Velocity (UPV) test results on mortar cubes with a fixed binder and varying sea sand content**
    As illustrated in Figure 9 (b) and (c), the introduction of 50$\%$ sea sand in the triple mix mortar cubes with mix designation TMF30G5S50, comprising 50$\%$ manufactured sand and 50$\%$ sea sand, results in only a 4.18$\%$ reduction in strength compared to TMF30G5R100 mortar mix with 100$\%$ river sand. This indicates that the combination of manufactured sand and sea sand replacement exhibits promising compressive strength parameters, showcasing potential advantages in mortar mix formulations. Additionally, the compressive strength attained by the TMF30G5S100 mix with 100$\%$ sea sand was just 12.35 N/$mm^2$, which was even 41.36$\%$ lesser than the mix TMF30G5R100, and 60.32$\%$ lesser than TMF30G5M100. Moreover, a similar effect was observed in the study conducted by Pranavan et al.^59^.
    From the Figure 9, (c) the UPV value of the mortar cubes with mix designation TMF30G5S50, comprising 50$\%$ M-sand $+$ 50$\%$ sea sand replacement was higher compared to the other mortar mixes with sea sand replacements from 50$\%$ to 90$\%$. From these observations made by the compressive strength test as well as the UPV test, the outcomes suggest that high-strength concrete can be produced using a 50$\%$ M-sand + 50$\%$ sea sand combination as the fine aggregate. This observation aligns with findings from the study^17^, suggesting that the presence of chloride ions positively influences the increase in compressive strength. Additionally, the study^59^ involved utilizing a blend of 50$\%$ M-sand and 50$\%$ sea sand as the fine aggregate for concrete production.
- **E]** **Mix proportions for M40-grade triple mix SCC incorporating sea sand**
  From the mortar study, 30$\%$ class F Fly Ash (FA), 5$\%$ Ground Granulated Blast Furnace Slag (GGBS), 65$\%$ of OPC, and a 50$\%$ sea sand replacement were chosen for the development of triple mix SCC. Additionally, from the mortar study on triple mix cubes with varying sea sand content, it was observed that cubes replaced with 10$\%$ sea sand by manufactured sand (TMF30G5S10) exhibited lower strength, while those replaced with 50$\%$ (TMF30G5S50) manufactured sand by sea sand demonstrated higher strength. Selecting cubes with both lower and higher strength with sea sand replacements, the development of M40 grade triple mix SCC followed. Additionally, triple mix SCCs with 100$\%$ river sand and 100$\%$ manufactured sand were included for comparative analysis. In total, six varieties of triple mix SCCs were considered for a comprehensive study encompassing fresh properties, mechanical properties, and durability.
  The mix proportions and mix designations obtained by the absolute volume method for M40-grade triple mix SCC incorporating sea sand and various fine aggregate replacements, after applying moisture corrections, are shown in Table 7.

**Table 7:** Mix proportions and the mix designations for M40 grade triple mix SCC incorporating sea sand.

Mix designation	Sample type	Cement kg/$m^3$	Fly Ash kg/$m^3$	GGBS kg/$m^3$	C.A. kg/$m^3$	M sand kg/$m^3$	Sea sand kg/$m^3$	River sand kg/$m^3$	Water lit/$m^3$	SP lit/$m^3$
TMSM100	Control mix	383.5	177	29.5	751.63	754.26	-	-	211.31	5.06
TMSM90	Sea sand variation	383.5	177	29.5	751.63	678.83	75.43	-	211.31	5.06
TMSM50	Sea sand variation	383.5	177	29.5	751.63	377.13	377.13	-	211.31	5.06
TMSR100	Comparative mix	383.5	177	29.5	782.64	-	-	781.86	208.34	5.06
TMSR90	Comparative mix	383.5	177	29.5	782.64	-	78.19	703.67	208.34	5.06
TMSR50	Comparative mix	383.5	177	29.5	782.64	-	390.93	390.93	208.34	5.06

Triple Mix Self-compacting concrete with OPC - 65$\%$, Fly ash - 30$\%$, and GGBS 5$\%$ - TMS, Manufactured sand - M, River sand - R.

TMSM100 - Triple mix SCC incorporating 100$\%$ Manufactured Sand, TMSM90 - Triple mix SCC incorporating 90$\%$ Manufactured Sand and 10$\%$ sea sand.

TMSM50 - Triple mix SCC incorporating 50$\%$ Manufactured Sand and 50$\%$ sea sand, TMSR100 - Triple mix SCC incorporating 100$\%$ River Sand.

**Figure 10:**
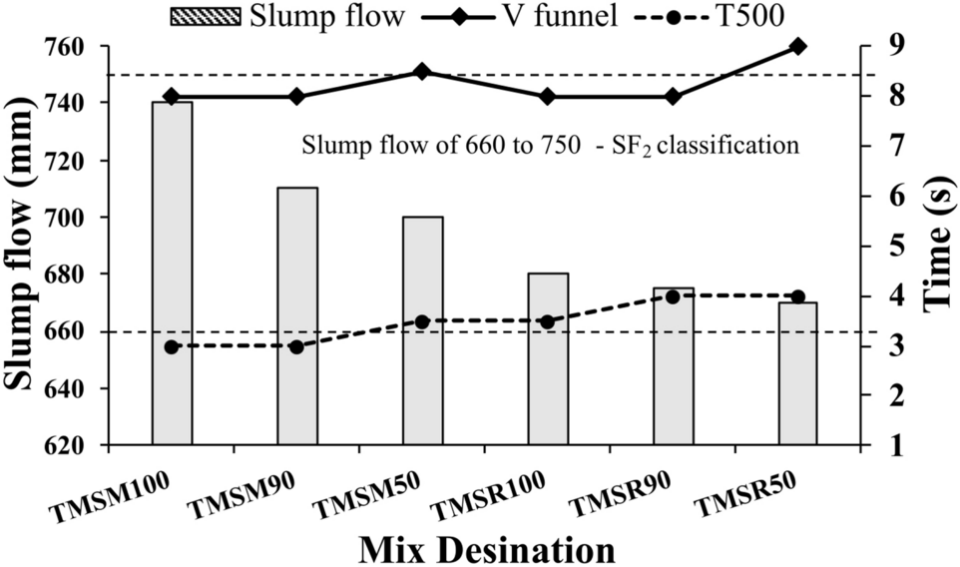
Fresh properties of SCC.

**Figure 11:**
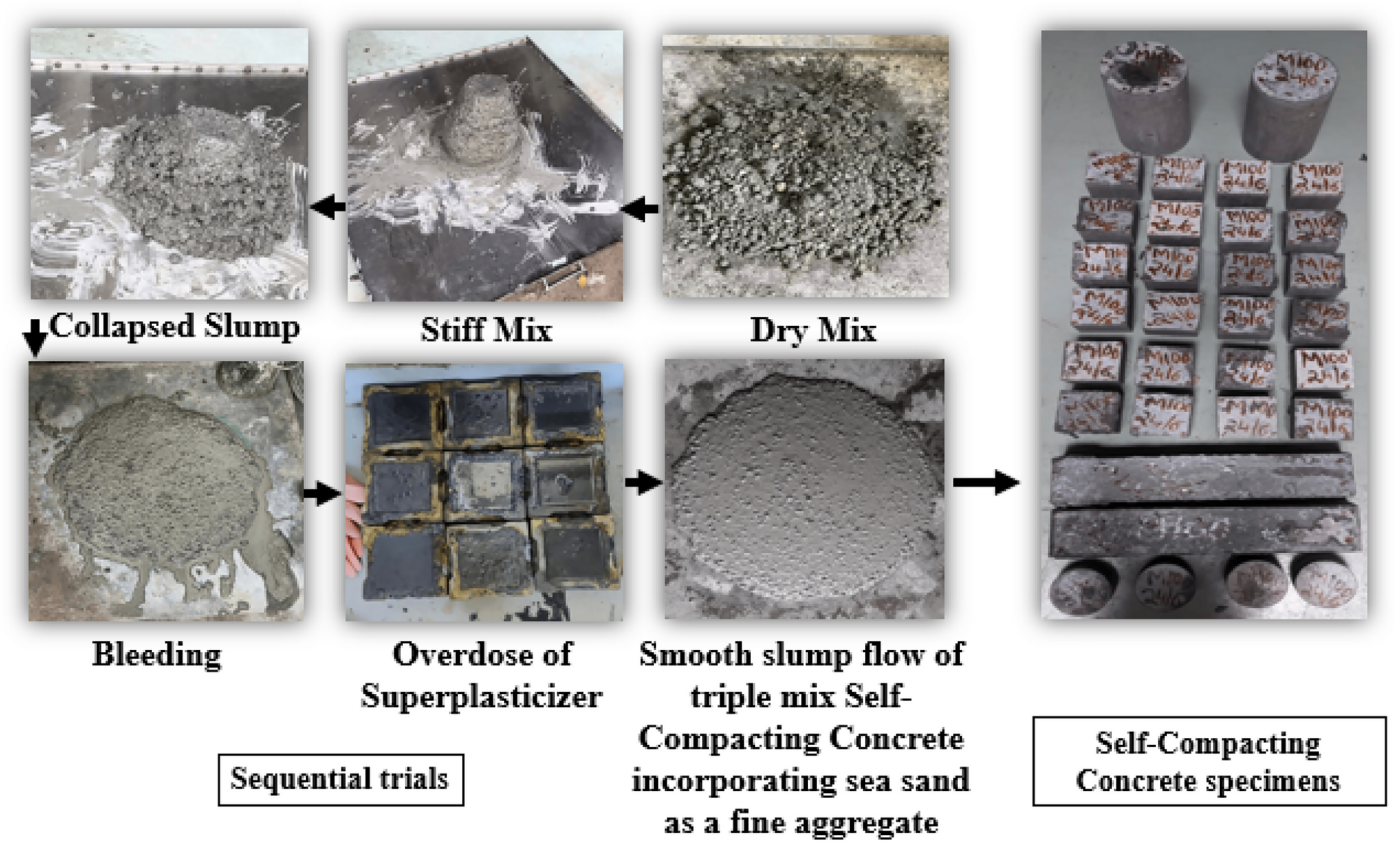
Sequential trials conducted to develop workable SCC.
  - $$\hbox {T}_{{500}}$$**time**
    From the study, SCC mixes with reduced slump exhibited extended $\hbox {T}_{{500}}$ times. However, all SCC mixes uniformly achieved a 500 mm diameter within less than 5 seconds, as illustrated in Figure 10. Also, the results were within the recommended range of 2 to 5 seconds as per EFNARC^12^ guidelines.
  - **V funnel test**
    The V funnel test was used to assess the filling capacity of the SCC mixes and to detect potential clogging issues if the unloading time is prolonged. The results of the V-funnel test, depicted in Figure 10, indicate that the V-funnel flow times for the SCC mixes range between 8 and 9 seconds, falling within the recommended range of 6 to 12 seconds for SCC.
- **G]** **Hardened concrete tests for studying mechanical properties of triple-mix SCC**
  - **Compressive strength**
    The Figure 12 displays the compressive strength results of various triple-mix SCC samples. As shown in Figure 12, the use of sea sand instead of river sand and manufactured sand does not significantly affect compressive strength.

TMSR90 - Triple mix SCC incorporating 90$\%$ River Sand and 10$\%$ sea sand, TMSR50 - Triple mix SCC incorporating 50$\%$ River Sand and 50$\%$ sea sand.
- **F]** **Tests conducted on the fresh properties of SCC**
  Figure 10 showcases the results obtained for the fresh properties of SCC.
  - **Slump flow**
    As the sea sand replacement rate in the SCC mixes increased, the slump-flow values in TMSM90 and TMSM50 decreased by 4.05$\%$ and 5.40$\%$, respectively, compared to TMSM100. In addition, the slump-flow values in TMSR90 and TMSR50 decreased by 0.74$\%$, and 1.47$\%$ respectively compared to TMSR100. Unprocessed sea sand adversely affected the workability of freshly mixed concrete, as reported by the study^17,60^. This impact was attributed to the higher fine particle content, passing through the 0.3 mm sieve, observed in Non-Treated Sea Sand (NSS) compared to Desalted Sea Sand (DSS). The elevated fine particle content tends to increase water demand in concrete, leading to reduced fluidity in fresh concrete. In a study conducted by Dang el al.^17^, concrete utilizing NSS exhibited lower initial slumps compared to those using DSS. A similar effect was observed in SCC mixes when sea sand was replaced with manufactured sand and river sand at 10$\%$ and 50$\%$ levels as portrayed in Figure 10. Furthermore, the rough texture of the sea sand, as analyzed by SEM analysis, reduced the slump flow of the SCC mixes when the sea sand was replaced at 10$\%$ and 50$\%$ (selected sea sand replacements which gave lower strength and higher strength from the mortar studies). Additionally, the Zone 1 river sand used in the study contains larger particles, which can hinder SCC movement and reduce its flowability, as shown in mix TMSR100. However, the addition of fly ash mitigates this effect as per the studies^17,61^ and demonstrated $\hbox {SF}_{{2}}$ slump flow classes, making them suitable for the construction of walls, piles, floors, and slabs according to EFNARC guidelines^12^. Sequential trials conducted to develop workable SCC are shown in Figure 11, which illustrates that the smooth slump flow obtained at the end of the trials showed no segregation or bleeding.

**Figure 12:**
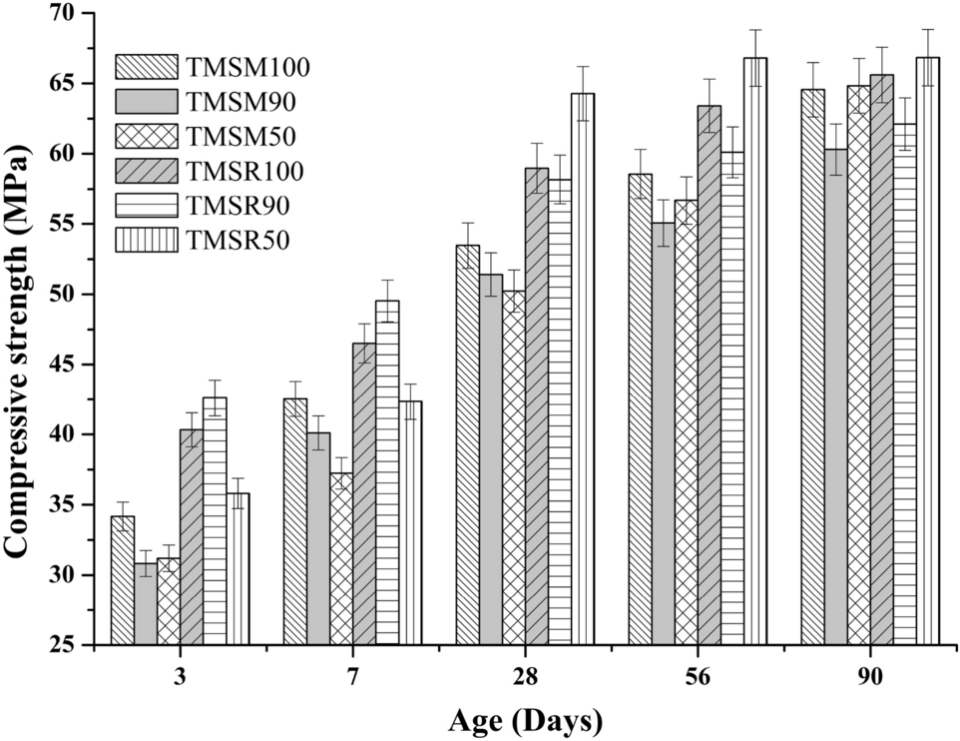
Compressive strength results of various triple-mix SCC samples.
    FA particles range from 1 to 100 $\mu$m, with those under 10 $\mu$m boosting early strength (till 28 days) and those between 10 and 45 $\mu$m aiding long-term strength (until 1 year)^62^. The presence of the highly soluble salts of the sea sand in the mixes TMSM50 and TMSR50 has quickened both cement setting and the pozzolanic reactivity of FA, resulting in increased 90$^{th}$ day compressive strength of TMSM50 and TMSR50 mixes. Also, chloride ions in sea sand interact with calcium hydroxide, resulting in the formation of calcium chloride, which significantly accelerates the hydration of Ordinary Portland Cement (OPC)^63^. These findings align with observations from the previous study^17^. This can be clarified by the enhancement in hydration processes at the later days and the generation of the better hydration products.
  - **Splitting tensile strength**
    The 28$^{th}$day splitting tensile strength of the mixes TMSM90 and TMSM50 was 0.32$\%$ more and 4.78$\%$ less, respectively, compared to TMSM100. Additionally, the mix TMSR90 showed a 0.003$\%$ increase in splitting tensile strength compared to TMSR100, while TMSR50 showed a 0.09$\%$ decrease in splitting tensile strength compared to TMSR100.
    The 56$^{th}$day splitting tensile strength of the mixes TMSM90 and TMSM50 was 7.52$\%$ and 11.42$\%$ lower, respectively, compared to TMSM100. Additionally, the mix TMSR90 showed a 6.52$\%$ decrease in splitting tensile strength compared to TMSM100, while TMSR50 showed a 7.93$\%$ reduction in splitting tensile strength compared to TMSR100.
    Furthermore, as illustrated in Figure 13, on the 90$^{th}$ day, the splitting tensile strength result of the SCC mixes TMSM90 and TMSM50 was 6.85$\%$, and 10.14$\%$ lower than that of TMSM100. Also, the mixes TMSM90, and TMSR50 showed 5.83$\%$ and 3.89$\%$ lesser splitting tensile strength, when compared to the mix TMSR100. Subsequently, the use of sea sand instead of river sand and manufactured sand does not significantly affect the splitting tensile strength of SCC.

**Figure 13:**
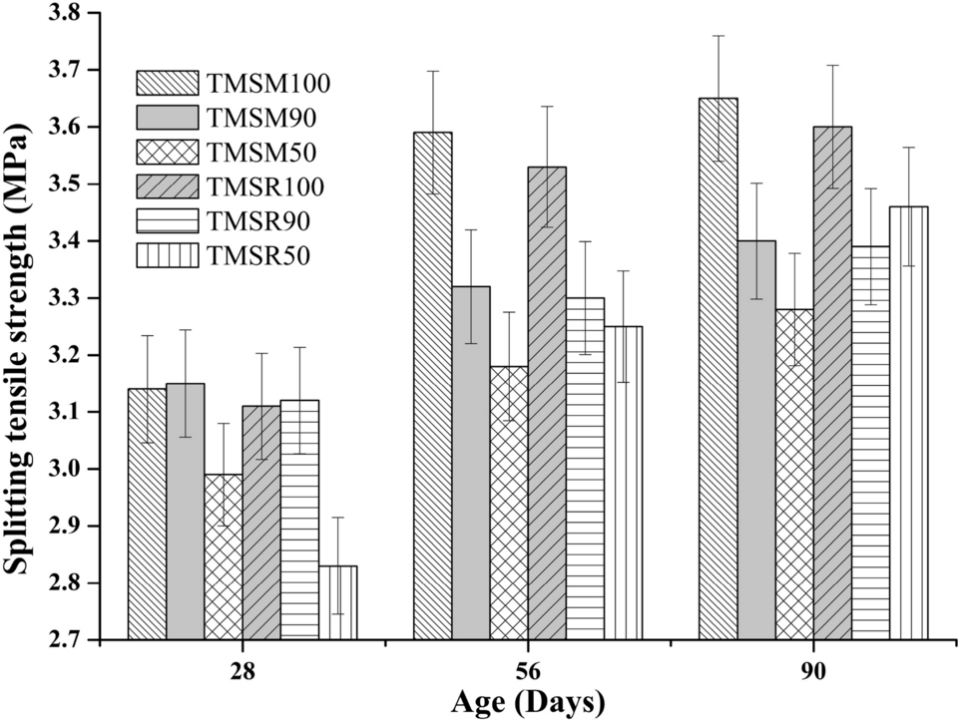
Splitting tensile strength results of various SCC mixes.
  - **Flexural strength of concrete**
    As shown in Figure 14, the flexural strengths of the SCC mixes improved as the curing times increased. After 90 days of curing, the flexural strengths of the SCC mixes ranged from 9 to 10.6 MPa.

**Figure 14:**
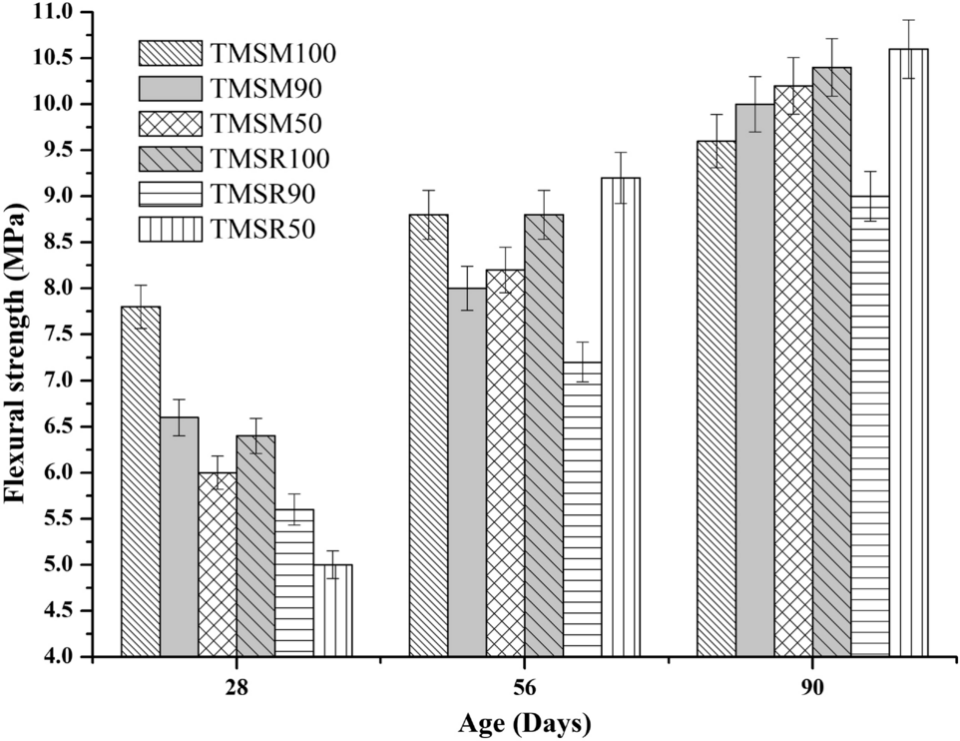
Flexural strength results of various SCC mixes.
    The 28$^{th}$day flexural strength of the mixes TMSM90 and TMSM50 was 15.38$\%$ and 23.08$\%$ less, respectively, compared to TMSM100. Additionally, the mix TMSR90 showed a 12.5$\%$ decrease in flexural strength compared to TMSR100, while TMSR50 showed a 21.88$\%$ decrease in flexural strength compared to TMSR100.
    The 56$^{th}$day flexural strength of the mixes TMSM90 and TMSM50 was 9.09$\%$ and 6.82$\%$ lower, respectively, compared to TMSM100. Additionally, the mix TMSR90 showed a 18.18$\%$ decrease in splitting tensile strength compared to TMSM100, while TMSR50 showed a 4.55$\%$ increase in flexural strength compared to TMSR100.
    After 90 days of curing, the mixes TMSM90 and TMSM50 showed increased flexural strengths of 4.17$\%$ and 6.25$\%$, respectively when compared to TMSM100. In contrast, TMSR90 exhibited 13.46$\%$ lower flexural strength, while TMSR50 had a 1.92$\%$ increase compared to TMSR100. This improvement can be attributed to the low porosity and good strength of both the cement paste matrix and the interfacial transition zone. Additionally, the rough texture of the sea sand particles provided a tight interlock between the binder and aggregate phases, contributing to the increased flexural strength. However, all SCC mixes replaced with sea sand showed an augmentation in flexural strength, excluding the SCC mix having 90$\%$ river sand and 10$\%$ sea sand, which was 13.46$\%$ lesser than the TMSR100.
- **H]** **Evaluation of the quality of SCC through a non-destructive approach**
  - **Ultrasonic Pulse Velocity (UPV)**
    SCC mixes demonstrated excellent and good quality grading, as shown in Figure 15.

**Figure 15:**
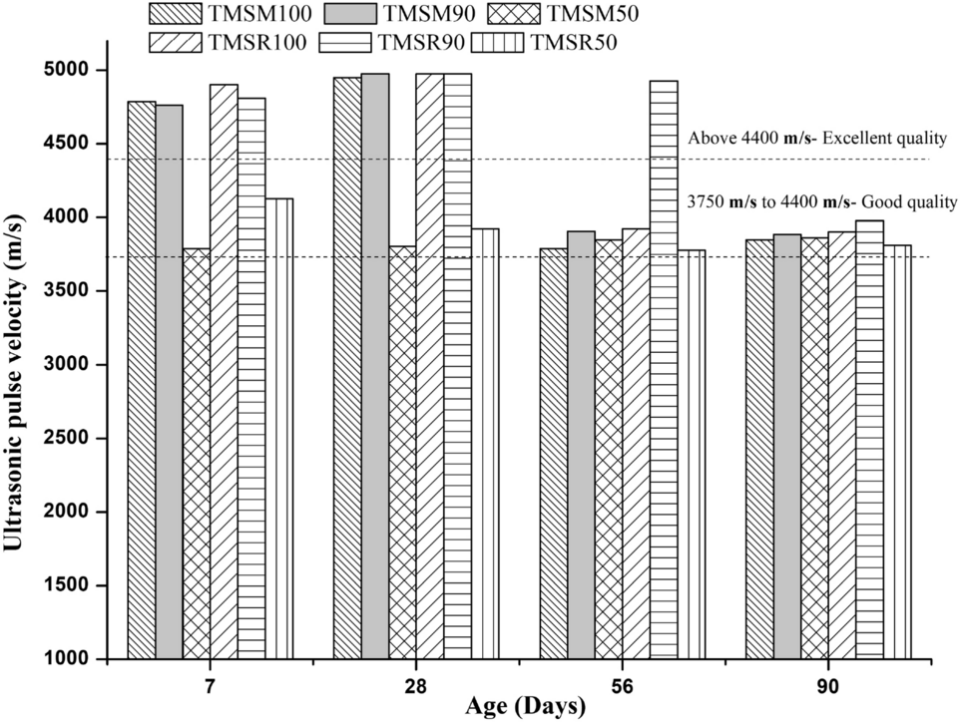
UPV values of various SCC mixes.
- **I]** **Microstructural analysis**
  - **Scanning Electron Microscope (SEM) analysis**
    From the SEM analysis, as depicted in Figure 16, it is evident that:
    - Figure 16 (a) TMSM100 (with 100$\%$ manufactured sand) exhibited higher porosity.
    - Figure 16 (b) TMSR100 (made with 100$\%$ river sand) and Figure 16 (a) TMSM100 did not display a densified matrix.
    - Figure 16 (c) TMSM90 (made with 90$\%$ M sand and 10$\%$ sea sand), and Figure 16 (d) TMSR90 (made with 90$\%$ river sand and 10$\%$ sea sand exhibited a densified matrix along with some pores.
    - Figure 16 (e) TMSM50 (made with 50$\%$ M sand and 50$\%$ sea sand) and Figure 16 (f) TMSR50 (made with 50$\%$ river sand and 50$\%$ sea sand) exhibited an even more compact microstructure with the formation of needle shaped C-S-H and plate-shaped Friedel’s salt.

**Figure 16:**
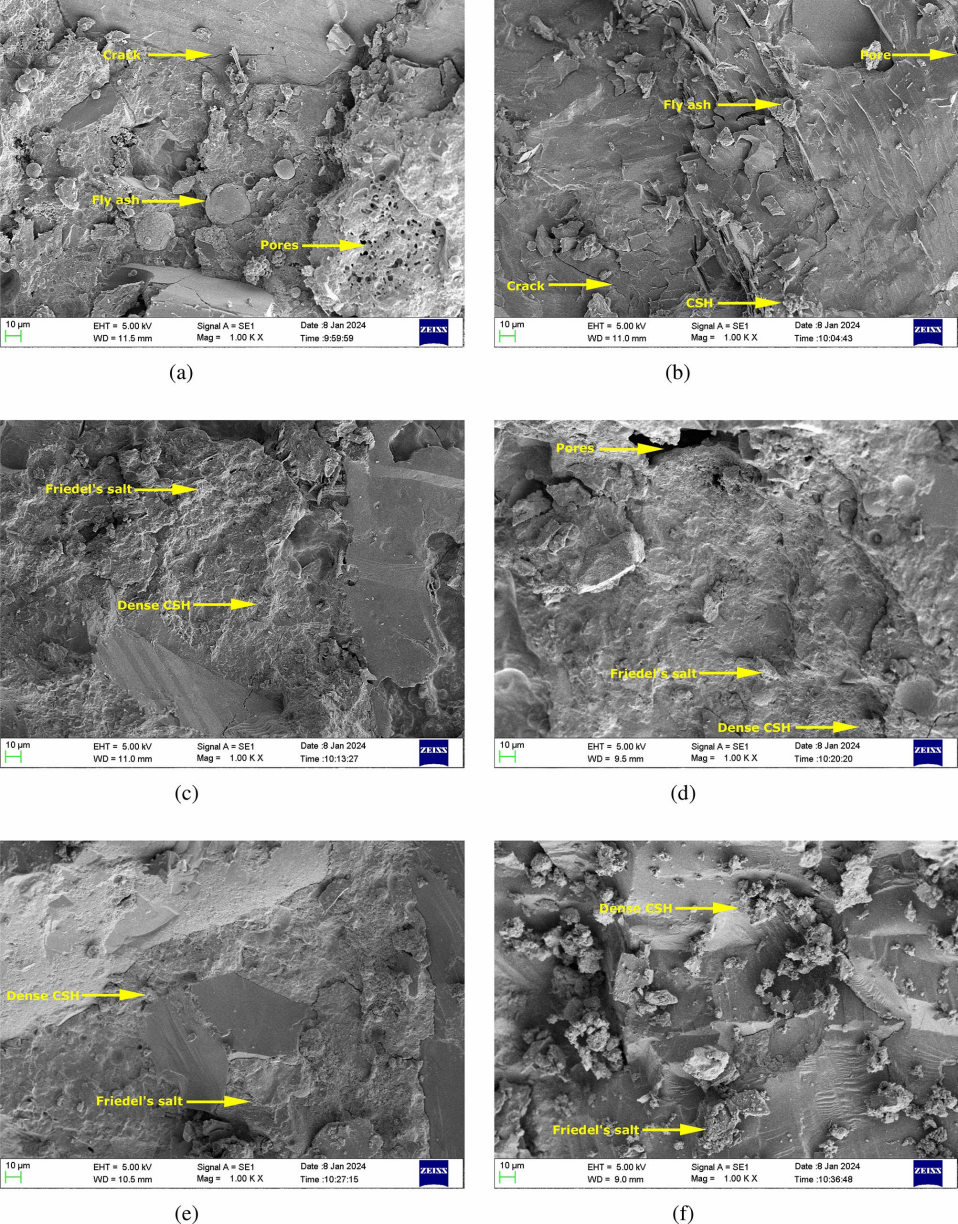
SEM images of various triple mix SCC at 90 days.
    The compactness observed was due to the interaction between chloride ions and the cement hydrate products. This interaction results in the formation of Friedel’s salt, refining the microstructure of SCC mixes that include sea sand^64^. Moreover, the incorporation of mineral admixtures resulted in decreased permeability in all the mixes. In contrast, Aluminum Oxide ($Al_2O_3$) present in Fly Ash (FA) and Blast Furnace Slag (BFS) has the potential to induce the formation of Friedel’s salt^64^. In addition, densified matrix of the TMSM50 and TMSR50 further implies the strong bond between the aggregate grains and the binder paste. Chloride binding in concrete involves both chemical and physical mechanisms. Chemical binding is related to Friedel’s salt, while physical binding depends on C-S-H content. Dense C-S-H formation from sea sand, confirmed by SEM images of the SCC mixes incorporating sea sand results in greater chloride binding^17^.
  - **Energy Dispersive Spectroscopy (EDS) analysis**
    Si, Al, and Ca were the primary elements detected in the EDS spectrum of the SCC specimens at a curing age of 90 days, as shown in Figure 17. The EDS analysis aimed to assess the impact of sea sand on the mechanical properties of concrete, with the results presented in Table 8. The introduction of sea sand contributed to a slight increase in Cl content, as indicated in Table 8. Numerous investigations have indicated that C-S-H formation during hydration is primarily driven by calcium (Ca) and silicate (Si) ions in the pore solution^65,66^. Additionally, for a water-to-cement ratio of 0.4, the Ca/Si remains steady at approximately 1.8 after 24 hours. A lower Ca/Si ratio results in a denser microstructure due to a stronger C-S-H network^66^. The Ca/Si ratio in mix TMSM100 was found to be 1.03, while TMSM50 showed a value of 0.75. This lower Ca/Si ratio contributed to the denser formation of the C-S-H gel, as observed in the SEM image.

**Figure 17:**
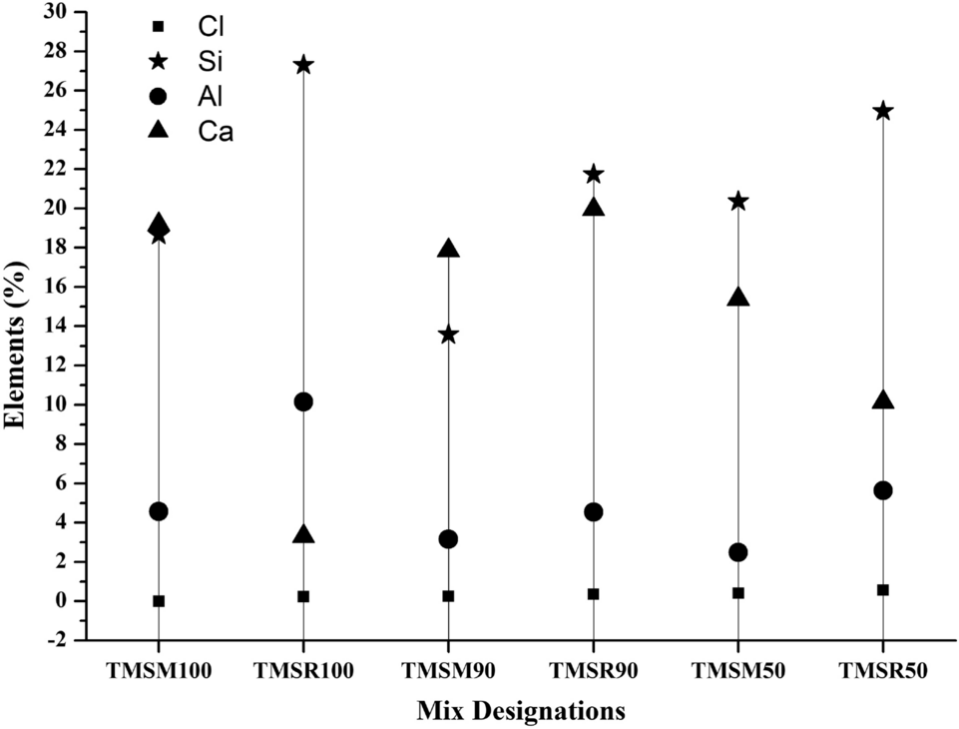
EDS values of various SCC mixes at 90 days.

**Table 8:** Presence of the elements expressed as a % by weight and their ratio in SCC mixes as per EDS analysis.

	Elements (expressed as a % by weight) and their ratio								
Mixes	Cl	Si	Al	Ca	Si+Al	Si/Al	Ca/(Si+Al)	Ca/Si	Ca/Al
TMSM100	0	18.64	4.57	19.18	23.21	4.08	0.83	1.03	4.20
TMSR100	0.23	27.30	10.15	3.30	37.45	2.69	0.09	0.12	0.33
TMSM90	0.25	13.58	3.16	17.85	16.74	4.30	1.07	1.31	5.65
TMSR90	0.36	21.74	4.53	19.95	26.27	4.80	0.76	0.92	4.40
TMSM50	0.40	20.36	2.48	15.38	22.84	8.21	0.67	0.75	6.20
TMSR50	0.56	24.95	5.63	10.14	30.58	4.43	0.33	0.41	1.80
    The combination of 50$\%$ sea sand with 50$\%$ manufactured sand performed better than mix TMSM100 and also outperformed the combination of 50$\%$ sea sand with 50$\%$ river sand as fine aggregates compared to mix TMSR100. However, the Ca/Si ratios of the SCC mixes incorporating sea sand were lower than 1.8, indicating that the extensive formation of C-S-H contributed to a quality microstructure and the production of additional hydration products through secondary hydration reactions, as evidenced by the SEM analysis, which accounts for the improved hardened properties and durability. Over time, the interaction between FA particles in the concrete mix and CH leads to the continuous formation of C-S-H gels. Thus, sea sand and SCMs prove to be viable alternatives for advanced concrete applications, addressing the growing demand for cement and river sand in construction.
  - **X-Ray Diffraction (XRD) analysis of SCC mixes**
    XRD analysis was used to examine hydration products in SCC mixes. Figure 18 displays XRD patterns of SCC samples at 90 days. Ettringite is evident with sharp peaks at 27.49$^{\circ }$ and 27.98$^{\circ }$ in all mixes. Quartz, identified by a peak at 26.60$^{\circ }$, indicates the presence of silicon dioxide-rich aggregates. Friedel’s salt formation is observed at 11.38$^{\circ }$ in SCC mixes with sea sand. Fly ash, renowned for its properties, contributes to ettringite formation^32^. Ettringite contributes to improved mechanical performance by generating a denser, less porous structure. Additionally, Friedel’s salt in the SCC mixes containing sea sand makes it more compact.

**Figure 18:**
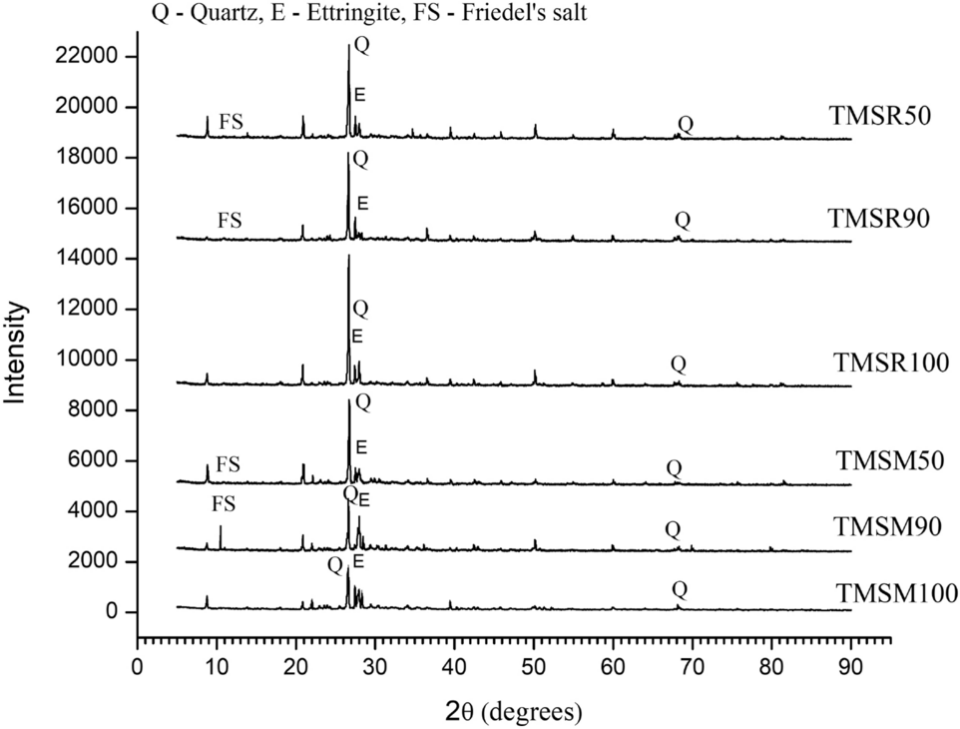
XRD spectra of SCC mixes at 90 days.
    The previous study showed that sulphate ion diffusion in concrete can produce expansive compounds like ettringite, gypsum, and salt crystals, causing significant damage. In Sea- Water Sea Sand Concrete (SWSSC), Friedel’s salt from $\hbox {Cl}^{-}$ consumes $\hbox {C}_{{3}}$A and CH, limiting their reaction with $\text {SO}_4^{2-}$ (sulphate) and reducing ettringite formation^55^. This phenomenon indicates that while ettringite formation was observed, it was not excess and does not cause damage.
- **J]** **Durability characteristics**
  - **Water absorption and density of SCC**
    The $28^{th}$ day water absorption of triple mix SCC are as shown in Figure 19. For TMSM100, water absorption was 4.36$\%$, while for TMSR100, it was 3.75$\%$. However, the mix TMSM90 showed a reduction in water absorption to 4.15$\%$, and for TMSR90, it was 2.31$\%$. Additionally, the mix TMSM50 reduced water absorption to 3.66$\%$, and TMSR50 further reduced water absorption to 2.79

**Figure 19:**
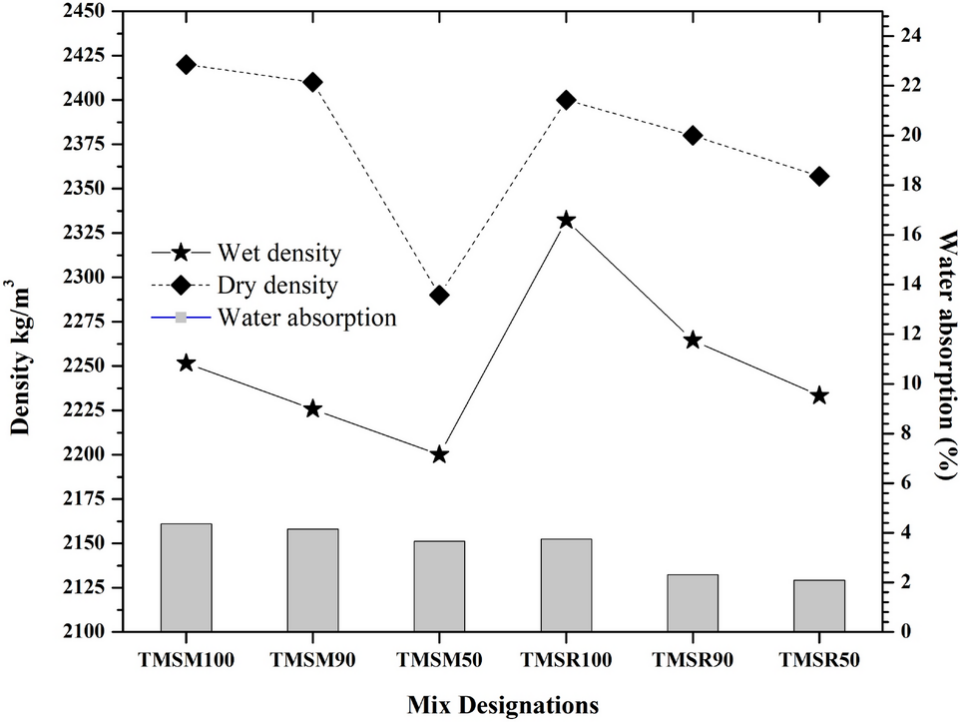
Density of SCC mixes and water absorption following immersion results of various SCC mixes.
    In accordance with the findings of the study^64^, the addition of sea sand reduced the porosity of the concrete. Compared to the 10$\%$ replacement of sea sand, a 50$\%$ replacement further reduced water absorption in the SCC mixes TMSM50 and TMSR50. This was attributed to the disconnected pore structure resulting from the production of Friedel’s salt^67^. Since water absorption is directly related to voids, its reduction is due to a denser concrete matrix. Additionally, lower water absorption indicates good compaction from the concrete’s own weight. Increased workability, especially with FA, enhances compaction. Pores, defects, and cracks in concrete increase water absorption, negatively impacting mechanical properties and durability^68^.
    1 $$\begin{aligned} Ca(OH)_2 + 2NaCl \rightarrow CaCl_2 + 2NaOH \end{aligned}$$
    2 $$\begin{aligned} 3CaO \cdot Al_2O_3 + CaCl_2 + 10H_2O \rightarrow 3CaO \cdot Al_2O_3 \cdot CaCl_2 \cdot 10H_2O \end{aligned}$$
    3 $$\begin{aligned} 3Ca(OH)_2 + CaCl_2 + Al_2O_3 + 7H_2O \rightarrow 3CaO \cdot Al_2O_3 \cdot CaCl_2 \cdot 10H_2O \end{aligned}$$
    The chloride ions present in the Non-desalted Sea Sand (NSS) can undergo reactions with tricalcium aluminate ($C_3A$) and Calcium Hydroxide ($CH$), resulting in the formation of Friedel’s salt ($3CaO \cdot Al_2O_3 \cdot CaCl_2 \cdot 10H_2O$)^64^. Conversely, Aluminum Oxide ($Al_2O_3$) Fly Ash (FA) and Blast Furnace Slag (BFS) can also contribute to the generation of Friedel’s salt. The formation process of Friedel’s salt is described by the provided equations 1, 2, 3^64^. However, SCC mixes incorporating M sand showed more water absorption compared to the SCC mixes with river sand.
    Furthermore, besides the reduction in water absorption, the mixes TMSM90 and TMSM50 exhibited a decrease in both wet and dry density compared to the mix TMSM100. Similarly, the SCC mixes TMSR90 and TMSR50 also demonstrated a decrease in both wet and dry density compared to the mix TMSR100, as illustrated in Figure 19. The reduction in the dry density of SCC with sea sand replacement helps to decrease the dead load of the structure. Consequently, this could lead to reductions in the cross-sectional dimensions of structural elements and potentially mitigate the risk of earthquake damage^68^.
  - **Sorptivity**
    SCC mixes incorporating sea sand (TMSM90, TMSM50, TMSR90, TMSR50) exhibited lower apparent porosity and sorptivity compared to SCC mixes containing 100$\%$ M sand and 100$\%$ river sand (TMSM100 and TMSR100), as illustrated in Figures 20 (a) and 20 (b).

**Figure 20:**
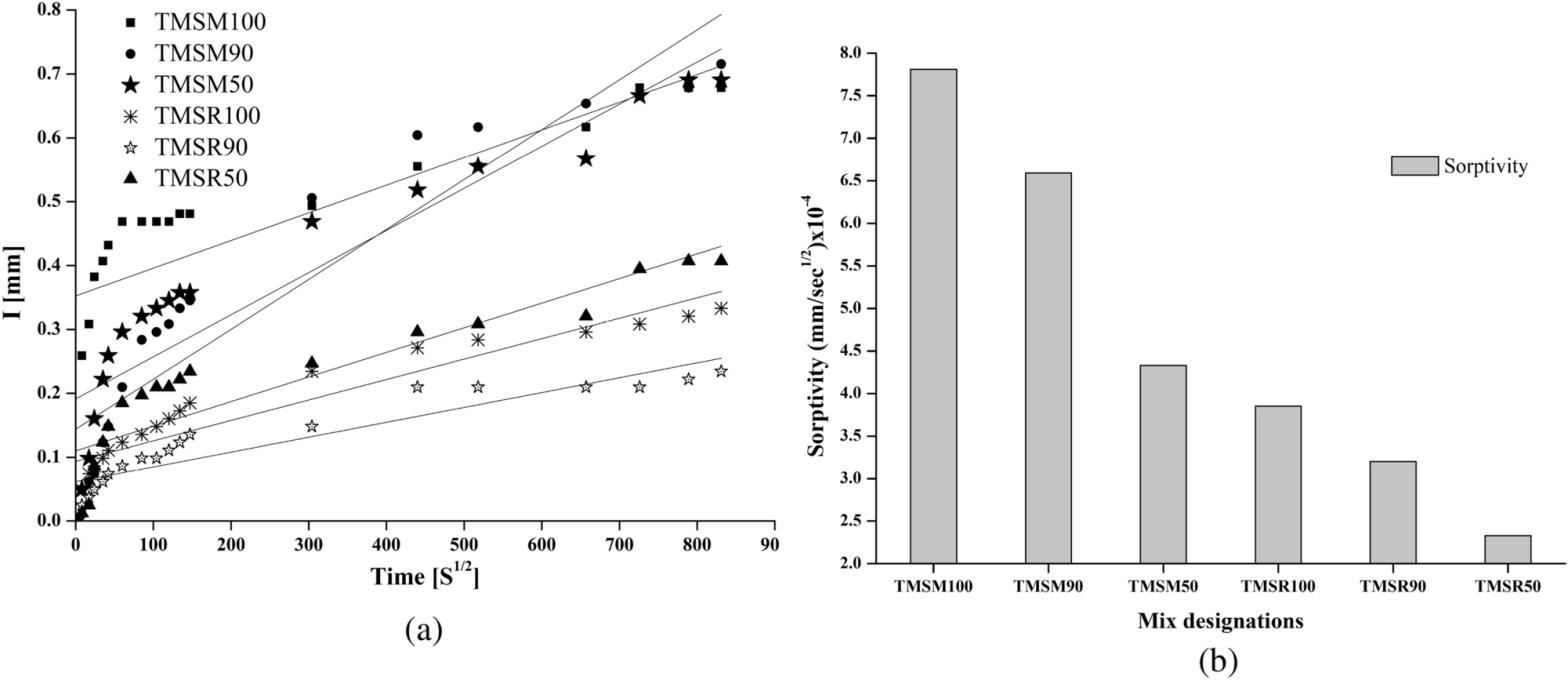
Rate of water absorption of SCC mixes.
    The sorptivity of the mixes TMSM90 and TMSM50 was 18.51$\%$ and 44.56$\%$ lower, respectively, compared to TMSM100. Additionally, the mix TMSR90 showed a 16.88$\%$ decrease in sorptivity compared to TMSM100, while TMSR50 showed a 39.48$\%$ lesser sorptivity compared to TMSR100.
    The decrease in sorptivity of SCC mixes with sea sand was attributed to chloride ions interacting with cement hydrate products, resulting in the formation of Friedel’s salt and microstructure enhancement in SCC mixes containing sea sand^37^. These results are consistent with previous studies^17,64^, which similarly reported lower apparent porosity and sorptivity in Non-desalted Sea Sand (NSS) concrete compared to Desalted Sea Sand (DSS) concrete. Additionally, high levels of soluble salts, such as sodium sulphate, calcium chloride, magnesium chloride, and sodium chloride, decrease the permeability of concrete. These salts accelerate the hydration of $\hbox {C}_{{3}}$S and $\hbox {C}_{{2}}$S in cement, releasing C$a^{2+}$ ions that form gypsum and calcium carbonate crystals, enhancing concrete’s properties compared to ordinary concrete^32^.
  - **Sulphuric acid resistance of hardened SCC mixes**
    Physical crystallization in TMSM50 and TMSR50 mixes refined their pore structure, improving sulphuric acid resistance, as illustrated in Figures 21 (a) and 21 (b).

**Figure 21:**
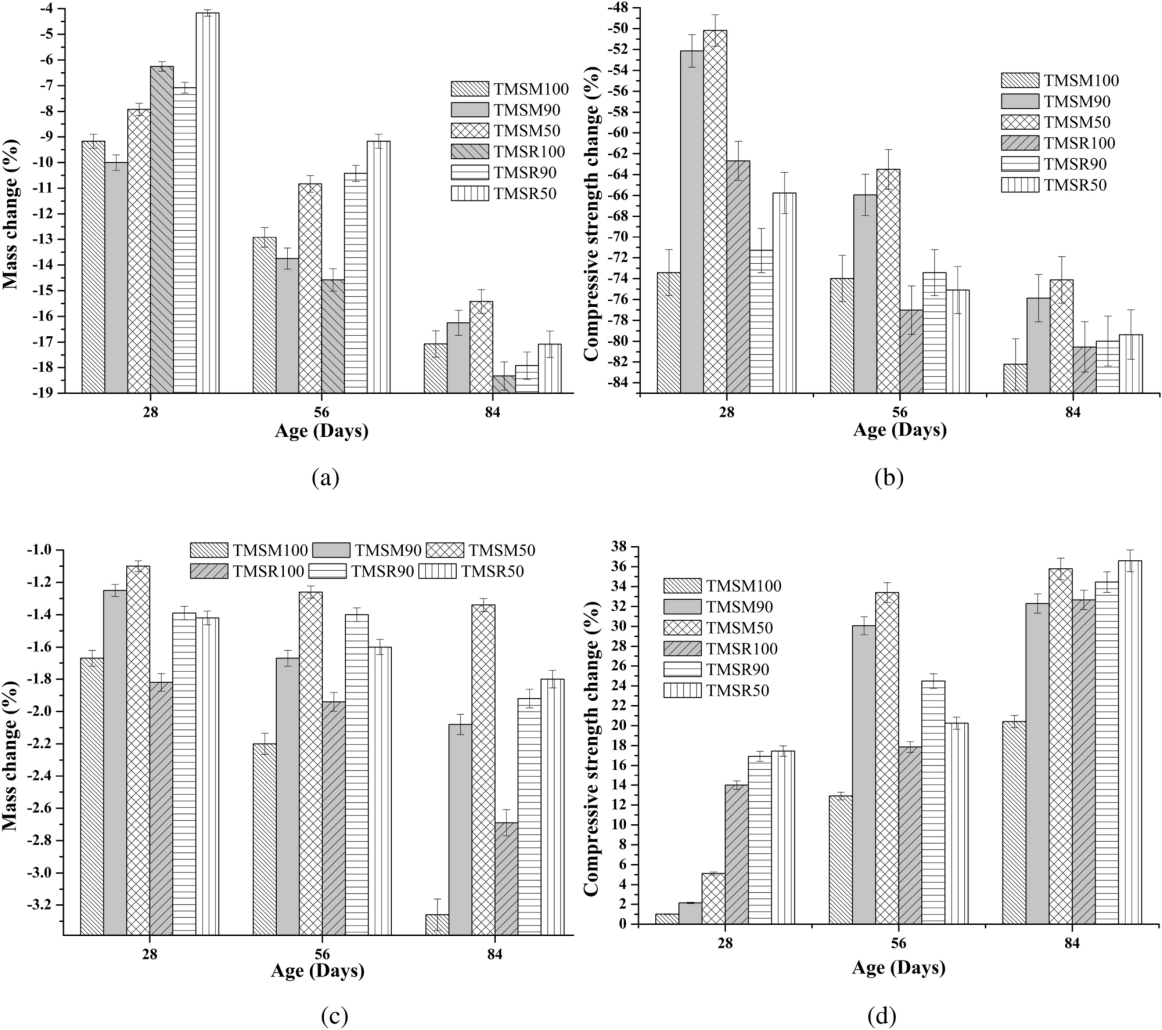
Hardened SCC specimens exposed to sulphuric acid environment and sulphate attack for 84 days.
  - **Sulphate attack test on hardened SCC mixes**
    Physical crystallization in TMSM50 and TMSR50 mixes refined their pore structure, improving sulphate resistance. Also, the formation of Friedel’s salt in these mixes suppressed expansion products, reducing $\hbox {SO}_{4}^{2-}$ (sulphate) erosion damage to the concrete. The mass loss of the SCC specimens exposed to the sulphate environment is shown in Figure 21 (c). Also, the compressive strength change of the SCC specimens exposed to the sulphate environment is shown in Figure 21 (d). Inferences from the sulphate attack test on the appearance of the specimens are given in Table 9.

**Table 9:** Inferences on the appearance of the specimen from sulphuric acid and sulphate attack tests^54,56^.

Inferences on the appearance of the specimens	Age		
	28	56	84
Sulphuric acid test inference on the appearance of the specimens	Smoothed corners	Deterioration of the cube shape	Extreme damage
Sulphate attack test inference on the appearance of the specimens	Good	Good	Good
  - **Rapid Chloride Permeability Test (RCPT)**
    Concrete with 30$\%$ fly ash replacement and a water-to-cement ratio of 0.5 demonstrated an extended initiation period against chloride attack, irrespective of the type of mixing water (seawater or tap water)^41,44^. The permeability of chloride ions through the SCC samples was found to be in the range of 100-1000 C. According to the ASTM C1202-22^57^ guidelines for interpreting results for chloride permeability, all the mixes showed very low chloride ion permeability. The use of mineral admixtures decreased the permeability to chloride ions, and the incorporation of sea sand in the mixes further reduced it, as shown in Figure  24. The mix TMSM90 showed 6$\%$ less charge passed in Coulombs compared to TMSM100. The mix TMSM50 showed 4$\%$ less charge passed in Coulombs compared to TMSM100. Additionally, the mix TMSR90 showed 31.1$\%$ less charge passed in Coulombs compared to TMSR100. The mix TMSR50 showed 66$\%$ less charge passed in Coulombs compared to TMSR100.
    Chloride ions accelerate the cement hydration process^69^. The introduction of seawater in the mix did not worsen chloride permeability due to the formation of Friedel’s Salt (FS)^70^. The same observation was noted in Figure 24. These observations align with the findings of the study^27^. In a previous investigation, the addition of Fly Ash (FA) substantially enhanced the resistance of Recycled Aggregate Seawater Sea Sand Concrete (RASSC) to chloride and sulphate erosion, as indicated in the study^27^.

**Figure 22:**
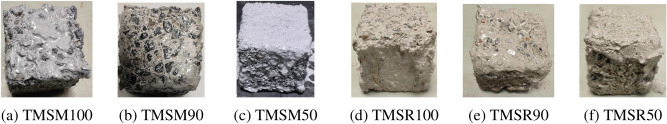
Hardened SCC specimens exposed to sulphuric acid for 84 days.

**Figure 23:**
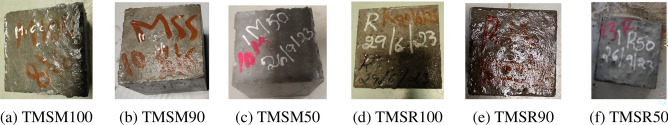
Hardened SCC specimens exposed to sulphate attack for 84 days.

**Figure 24:**
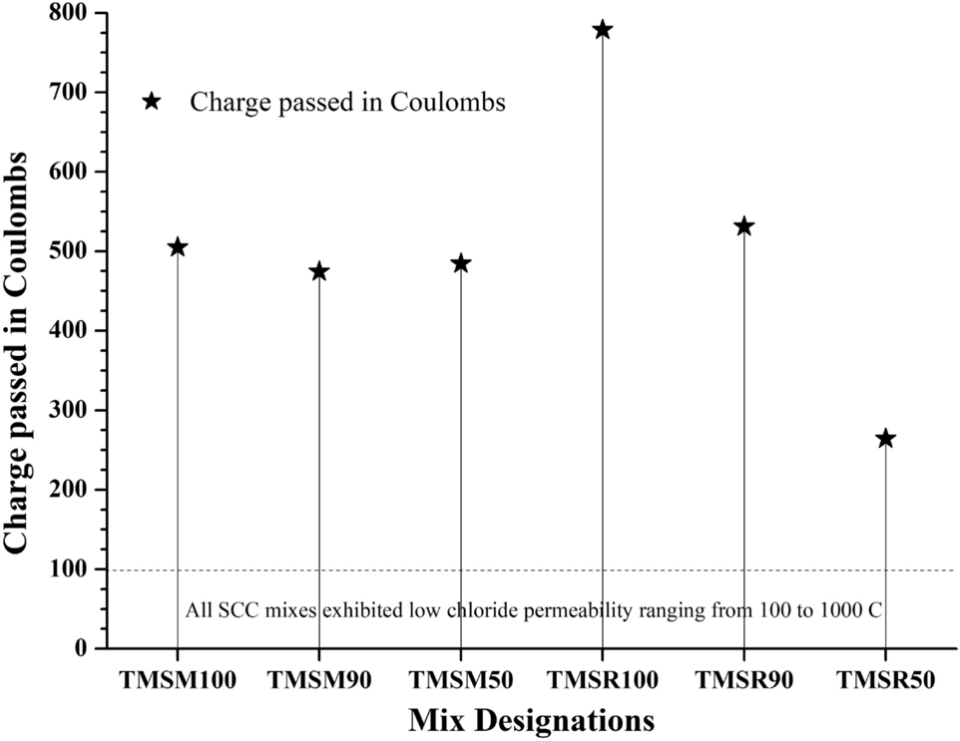
RCPT results of various SCC mixes.

## Conclusions

- **A]** SCC mixes TMSM50 and TMSR50 achieved the highest compressive strength on the 90$^{th}$ day, due to improved hydration processes and better hydration products.
- **B]** The rough surface texture of the sea sand particles provided a tight interlock between the binder and aggregate phases, contributing to increased flexural strength.
- **C]** Incorporating sea sand reduced concrete porosity and sorptivity, resulting in decreased water absorption in SCC mixes with sea sand.
- **D]** SCMs and sea sand reduced chloride ion permeability in RCPT tests, which can be attributed to microstructure refinement through accelerated CSH and Friedel’s salt formation, as observed in the SEM analysis.
- **E]** Physical crystallization in TMSM50 and TMSR50 mixes refined their pore structure, improving sulphate resistance. Additionally, the formation of Friedel’s salt in these mixes suppressed expansion products, reducing $\hbox {SO}_{4}^{2-}$ erosion damage to the concrete.
- **F]** The developed SCC mixes incorporating sea sand improved the durability of SCC through both chemical and physical chloride binding mechanisms.
- **G]** UPV test results indicated that all the SCC mixes demonstrated either excellent or good-quality grading of the concrete.
- **H]** The good quality and lower dry density of SCC with sea sand reduce the dead load of structures, potentially allowing for smaller cross-sectional dimensions of structural elements.

## Future perspectives

- **A]** The inclusion of sea sand as a potential substitute for river sand, along with the incorporation of SCMs mentioned in the article, provides diversified options for researchers and industry professionals, facilitating the adoption of sustainable construction practices.
- **B]** Future investigations should prioritize thoroughly assessing the extended durability of SCC incorporating sea sand as a fine aggregate, particularly in conjunction with Fiber Reinforced Polymer (FRP) materials, textile reinforcements, coated rebars, stainless steel bars, Aluminum Metal Matrix Composites (AMMCs)^71^, and the application of Corrosion Inhibiting Admixture (CIA) in reinforced concrete.

## Supplementary Information

Supplementary Information.

## Data Availability

The datasets used and analyzed during the current study are available from the corresponding author upon reasonable request.

## References

[1] Saranya, T., Ambily, P.S., & Raj, B. “Studies on the utilization of alternative fine aggregate in geopolymer concrete,” in *National Conference on Structural Engineering and Construction Management*. Springer, pp. 851–859 (2019). 10.1007/978-3-030-26365-2_78

[2] Gupta, N., Siddique, R., & Belarbi, R. “Sustainable and greener self-compacting concrete incorporating industrial by-products: A review,” *Journal of Cleaner Production*, p. 124803, (2020).10.1016/j.jclepro.2020.124803

[3] Das, S.K., Mishra, J., Singh, S.K., Mustakim, S.M., Patel, A., Das, S.K., & Behera, U. “Characterization and utilization of rice husk ash (rha) in fly ash–blast furnace slag based geopolymer concrete for sustainable future,” *Materials Today: Proceedings*, (2020). 10.1016/j.matpr.2020.02.870

[4] R. K, “Performance study on partial replacement of with fly ash, rice husk ash and limestone powder in hybrid fiber reinforced concrete,” 2018, [Online] Available: http://hdl.handle.net/10603/261936; Last accessed date: 20-October-2020.

[5] Torres-Carrasco, M. et al. Critical aspects in the handling of reactive silica in cementitious materials: Effectiveness of rice husk ash vs nano-silica in mortar dosage. *Construction and Building Materials* **223**, 360–367. 10.1016/j.conbuildmat.2019.07.023 (2019).

[6] Afshar, A. et al. Corrosion resistance evaluation of rebars with various primers and coatings in concrete modified with different additives. *Construction and Building Materials ***262**, 120034. 10.1016/j.conbuildmat.2020.120034 (2020).

[7] Ramachandra, T., Vinay, S. & Chandran, M. S. Quantification of annual sediment deposits for sustainable sand management in aghanashini river estuary. *Journal of environmental management ***206**, 1263–1273. 10.1016/j.jenvman.2017.07.060 (2018). 28807516 10.1016/j.jenvman.2017.07.060

[8] Ramachandra, T., Vinay, S., & Chandran, M.S. Sand mining and its imapact on ecology of aghanashini estuary uttara kannada district, karnataka. [Online] Available: http://wgbis.ces.iisc.ernet.in/biodiversity/sahyadri_enews/newsletter/Issue59/posters/11-Sand-mining-aghanashini.pdf; Last accessed date: 15-December-2020.

[9] Guo, M. et al. Characterization of the mechanical properties of eco-friendly concrete made with untreated sea sand and seawater based on statistical analysis. *Construction and Building Materials ***234**, 117339. 10.1016/j.conbuildmat.2019.117339 (2020).

[10] Gao, Y. et al. Blast responses of one-way sea-sand seawater concrete slabs reinforced with bfrp bars. *Construction and Building Materials ***232**, 117254. 10.1016/j.conbuildmat.2019.117254 (2020).

[11] *IS 456:2000, Code of practice for the plain and reinforced concrete*. Bureau of Indian Standards, India.

[12] *Specification and Guidelines for Self-Compacting Concrete*. EFNARC, (2005).

[13] Yang, S., Zang, C., Xu, J. & Fan, G. Determination of fracture parameters of seawater sea sand concrete based on maximum fracture load. *Journal of Materials in Civil Engineering ***32**(1), 04019315. 10.1061/(ASCE)MT.1943-5533.0002981 (2020).

[14] Li, Y., Zhao, X., Singh, R. R. & Al-Saadi, S. Experimental study on seawater and sea sand concrete filled gfrp and stainless steel tubular stub columns. *Thin-Walled Structures ***106**, 390–406. 10.1016/j.tws.2016.05.014 (2016).

[15] Li, L., Zeng, L., Li, S. & Liu, F. Shear capacity of sea sand concrete beams with polymer bars. *Proceedings of the Institution of Civil Engineers-Structures and Buildings ***172**(4), 237–248. 10.1680/jstbu.16.00164 (2019).

[16] Sayed, U. et al. Bamboo stick diameter, volume and aspect ratios effect on the compressive behavior of bamboo sticks reinforced concrete mixed with sea sand and seawater. *Construction and Building Materials ***369**, 130437 (2023).

[17] Dang, V. Q., Ogawa, Y., Bui, P. T. & Kawai, K. Effects of chloride ion in sea sand on properties of fresh and hardened concrete incorporating supplementary cementitious materials. *Journal of Sustainable Cement-Based Materials ***11**(6), 439–451 (2022).

[18] Kryzhanovskyi, V., Avramidou, A., Orlowsky, J. & Spyridis, P. Self-compacting high-strength textile-reinforced concrete using sea sand and sea water. *Materials ***16**(14), 4934 (2023). 37512208 10.3390/ma16144934 PMC10381865

[19] Sang, N. T. et al. Performances of eco-fine-grained concrete containing saline sand as partial fine aggregate replacement. *Journal of Applied Science and Engineering ***24**(4), 527–539. 10.6180/jase.202108_24(4).0009 (2021).

[20] *IS 10262:2019, Concrete Mix Proportioning guidelines*. Bureau of Indian Standards, India.

[21] Bhatawdekar, R. M., Singh, T. N., Tonnizam Mohamad, E., Armaghani, D. J., & Binti Abang Hasbollah, D. Z. “River sand mining vis a vis manufactured sand for sustainability,” in *Proceedings of the International Conference on Innovations for Sustainable and Responsible Mining: ISRM 2020-Volume 1*. Springer, 2021, pp. 143–169.

[22] Gallagher, L., & Peduzzi, P. “Sand and sustainability: Finding new solutions for environmental governance of global sand resources,” (2019).

[23] Dinakar, P., Reddy, M. K. & Sharma, M. Behaviour of self compacting concrete using portland pozzolana cement with different levels of fly ash. *Materials & Design ***46**, 609–616 (2013).

[24] G. A, “An experimental study on impact strength of self compacting concrete using admixtures,” 2016, [Online] Available: http://hdl.handle.net/10603/181332; Last accessed date: 20-October-2020. http://hdl.handle.net/10603/181332

[25] Kavyateja, B. V., Jawahar, J. G., & Sashidhar, C. “Effectiveness of alccofine and fly ash on mechanical properties of ternary blended self compacting concrete,” *Materials Today: Proceedings*, (2020). 10.1016/j.matpr.2020.03.152

[26] Divitkumar, R., Ganesh, B., Shrestha, J., Pandit, B.P., Chaulagain, A., & Rawat, B. “Rheology of sustainable self compacting concrete with triple blend cementitious materials,” in *International Conference on Structural Engineering and Construction Management*. Springer, (2021), pp. 905–919. 10.1007/978-3-030-80312-4_77

[27] Lu, Z. et al. Recycled aggregate seawater-sea sand concrete and its durability after immersion in seawater. *Journal of Building Engineering ***65**, 105780 (2023).

[28] Dai, J.-G., Bai, Y.-L. & Teng, J. Behavior and modeling of concrete confined with frp composites of large deformability. *Journal of composites for construction ***15**(6), 963–973. 10.1061/(ASCE)CC.1943-5614.0000230 (2011).

[29] Liu, W. et al. Carbonation of concrete made with dredged marine sand and its effect on chloride binding. *Construction and Building Materials ***120**, 1–9 (2016). https://www.sciencedirect.com/science/article/pii/S0950061816307309

[30] Pungercar, V., & Musso, F. “Salt as a building material: Current status and future opportunities,” *The plan Journal*.

[31] Tjaronge, M.W., Irmawaty, R., Adisasmita, S.A., Amiruddin, A., & Hartini, “Compressive strength and hydration process of self compacting concrete (scc) mixed with sea water, marine sand and portland composite cement,” *Advanced Materials Research *, vol. 935, pp. 242–246, (2014)

[32] Pan, D. et al. Study of the influence of seawater and sea sand on the mechanical and microstructural properties of concrete. *Journal of Building Engineering ***42**, 103006. 10.1016/j.jobe.2021.103006 (2021).

[33] Mohan, A. & Mini, K. Strength and durability studies of scc incorporating silica fume and ultra fine ggbs. *Construction and Building Materials ***171**, 919–928. 10.1016/j.conbuildmat.2018.03.186 (2018).

[34] Thomas, J. & Ramaswamy, A. Mechanical properties of steel fiber-reinforced concrete. *Journal of materials in civil engineering ***19**(5), 385–392 (2007).

[35] Li, H., Farzadnia, N. & Shi, C. The role of seawater in interaction of slag and silica fume with cement in low water-to-binder ratio pastes at the early age of hydration. *Construction and Building Materials ***185**, 508–518 (2018). https://www.sciencedirect.com/science/article/pii/S095006181831763X

[36] Zhao, Y., Hu, X., Shi, C., Zhang, Z. & Zhu, D. A review on seawater sea-sand concrete: Mixture proportion, hydration, microstructure and properties. *Construction and Building Materials ***295**, 123602 (2021).

[37] Sindhurashmi, B., Nayak, G., Adesh, N., Rao, V. & Dubey, S. P. Incorporating sea sand into self-compacting concrete: a systematic review. *Discover Applied Sciences ***6**(4), 194. 10.1007/s42452-024-05826-0 (2024). 10.1038/s41598-024-75613-9 PMC11494173 39433953

[38] L. D. L. F. N. A. S. P. Y. T. Ebead, Usama, “A review of recent advances in the science and technology of seawater-mixed concrete,” *Cement and Concrete Research *, vol. 152, (2022).

[39] Wang, Y. et al. Experimental study on foci development in mortar using seawater and sand. *Materials ***12**(11), 1799 (2019). 31163604 10.3390/ma12111799 PMC6600738

[40] *IS 269:2015, Ordinary portland cement specification*. Bureau of Indian Standards, India.

[41] Rathnarajan, S., & Sikora, P. “Seawater-mixed concretes containing natural and sea sand aggregates–a review,” *Results in Engineering*, p. 101457, (2023).

[42] Yang, S. et al. A closed-form fracture model to predict tensile strength and fracture toughness of alkali-activated slag and fly ash blended concrete made by sea sand and recycled coarse aggregate. *Construction and Building Materials ***300**, 123976 (2021).

[43] Sang, N. T. et al. Applicability of concrete containing the binary and ternary system of binder materials under natural marine environment. *Journal of Applied Science and Engineering ***25**(5), 881–891 (2022).

[44] Otsuki, N., Nishida, T., Yi, C., Nagata, T., & Ohara, H. “Effect of blast furnace slag powder and fly ash on durability of concrete mixed with seawater,” (2014).

[45] *ASTM C642-21, Standard Test Method for Density, Absorption, and Voids in Hardened Concrete*. ASTM International, [Online] Available: https://compass.astm.org/document/?contentCode=ASTM%7CC0642-21%7Cen-US; Last accessed date: 6-October-2023.

[46] J. 206-2010, “Technical code for application of sea sand concrete,” (2010).

[47] Karthikeyan, M., & Nagarajan, V. “Study on sea sand as fine aggregate replacement in concrete,” 2018, [Online] Available: http://hdl.handle.net/10603/233682; Last accessed date: 20-October-2020.

[48] *IS 516, Part 1, Testing of strength of hardened concrete*. Bureau of Indian Standards, India.

[49] *IS 5816:2018, Method of test splitting tensile strength of concrete*. Bureau of Indian Standards, India.

[50] Wang, Z. et al. Durability study on interlaminar shear behaviour of basalt-, glass-and carbon-fibre reinforced polymer (b/g/cfrp) bars in seawater sea sand concrete environment. *Construction and Building Materials ***156**, 985–1004. 10.1016/j.conbuildmat.2017.09.045 (2017).

[51] *IS 516, Part 5, Non-destructive testing of concrete*. Bureau of Indian Standards, India.

[52] BS 1881–122. *Method for determination of water absorption* (British Standards Institution, London, 1983).

[53] *ASTM C1585-20, Standard Test Method for Measurement of Rate of Absorption of Water by Hydraulic-Cement Concretes*. ASTM International.

[54] *ASTM C1898-20, Standard test methods for determining the chemical resistance of concrete products to acid attack*. ASTM International, [Online] Available: https://compass.astm.org/document/?contentCode=ASTM%7CC1898-20%7Cen-US; Last accessed date: 17-May-2024.

[55] Zhang, D. et al. Comparative analysis of sulfate resistance between seawater sea sand concrete and freshwater desalted sea sand concrete under different exposure environments. *Construction and Building Materials ***416**, 135146. 10.1016/j.conbuildmat.2024.135146 (2024).

[56] *ASTM C267-20, Standard test methods for chemical resistance of mortars, grouts, and monolithic surfacings and polymer concretes*. ASTM International, [Online] Available: https://compass.astm.org/document/?contentCode=ASTM%7CC0267-20%7Cen-US; Last accessed date: 17-May-2024.

[57] *ASTM C1202-22, Standard Test Method for Electrical Indication of Concrete’s Ability to Resist Chloride Ion Penetration*. ASTM International, [Online] Available: https://compass.astm.org/document/?contentCode=ASTM%7CC1202-22%7Cen-US; Last accessed date: 6-October-2023.

[58] *IS 12269:2013, Ordinary Portland Cement 53 grade specification*. Bureau of Indian Standards, India.

[59] Pranavan, S. & Srinivasan, G. Investigation on behaviour of m-sand and sea sand based concrete. *Materials Today: Proceedings ***45**, 7079–7085 (2021).

[60] Natarajan, S., Neelakanda Pillai, N. & Murugan, S. Experimental investigations on the properties of epoxy-resin-bonded cement concrete containing sea sand for use in unreinforced concrete applications. *Materials ***12**(4), 645. 10.3390/ma12040645 (2019). 30791680 10.3390/ma12040645 PMC6416622

[61] Liu, J. et al. Effects of w/b ratio, fly ash, limestone calcined clay, seawater and sea-sand on workability, mechanical properties, drying shrinkage behavior and micro-structural characteristics of concrete. *Construction and Building Materials ***321**, 126333 (2022).

[62] Bapat, J. D. *Mineral admixtures in cement and concrete*. Taylor and Francis Group, (2013).

[63] Phuc, D. V., & Khoa, N. T. “Effect of ground granulated blast furnace slag and fly ash on mechanical properties and sulfate attack resistance of fine-grained concrete using sea sand,” in *2022 7th National Scientific Conference on Applying New Technology in Green Buildings (ATiGB)*. IEEE, pp. 183–188 (2022).

[64] Dang, V. Q., Ogawa, Y., Bui, P. T. & Kawai, K. Effects of chloride ions on the durability and mechanical properties of sea sand concrete incorporating supplementary cementitious materials under an accelerated carbonation condition. *Construction and Building Materials ***274**, 122016 (2021). https://www.sciencedirect.com/science/article/pii/S0950061820340204

[65] Scrivener, K., Snellings, R., Lothenbach, B., & Press, C. *A practical guide to microstructural analysis of cementitious materials*. Crc Press Boca Raton, FL, USA:, vol. 540 (2016).

[66] Sumukh, E. P., Das, B. B. & Barbhuiya, S. Effect of iron ore and copper ore tailings on engineering properties and hydration products of sustainable cement mortar. *Advances in Civil Engineering Materials ***13**(1), 50–75 (2024).

[67] Saleh, S., Mahmood, A. H., Hamed, E. & Zhao, X.-L. The mechanical, transport and chloride binding characteristics of ultra-high-performance concrete utilising seawater, sea sand and scms. *Construction and Building Materials ***372**, 130815 (2023).

[68] Şenol, A. F. & Karakurt, C. High-strength self-compacting concrete produced with recycled clay brick powders: Rheological, mechanical and microstructural properties. *Journal of Building Engineering ***88**, 109175. 10.1016/j.jobe.2024.109175 (2024).

[69] Wang, D., Gong, Q., Yuan, Q. & Luo, S. Review of the properties of fiber-reinforced polymer-reinforced seawater-sea sand concrete. *Journal of Materials in Civil Engineering ***33**(10), 04021285 (2021).

[70] Cheng, S., Shui, Z., Sun, T., Huang, Y., & Liu, K. “Effects of seawater and supplementary cementitious materials on the durability and microstructure of lightweight aggregate concrete,” *Construction and Building Materials*, pp. 1081–1090, 2018. https://www.sciencedirect.com/science/article/pii/S0950061818323663

[71] Amirtharaj, J., & Mariappan, M. “Exploring the potential uses of aluminium metal matrix composites (ammcs) as alternatives to steel bar in reinforced concrete (rc) structures-a state of art review,” *Journal of Building Engineering*, p. 108085, (2023).

