# Supplementary material for: Mock-up pragmatic study on the impact performance of self-compacting concrete incorporating sea sand
Source: Sci Rep. 2024 Oct 21;14:24744. doi: 10.1038/s41598-024-75613-9 (PMC11494173; doi:10.1038/s41598-024-75613-9)
Supplement: Supplementary file 1 — Supplementary Information. [file 41598_2024_75613_MOESM1_ESM.pdf]

Table 1: Tests conducted for the pragmatic study

| Sl. No. | Tests conducted | Sample used | Instrument/Methods/Standards |
|---|---|---|---|
| 1 | Particle size distribution | OPC, FA, GGBS | Malvern Mastersizer 3000 |
| 2 | Specific gravity | OPC, FA, GGBS | Le Chatelier's flask method as per IS 4031: Part 11 |
| 3 | Specific gravity | Fine aggregates (*Sea sand, river sand, manufactured sand) and coarse aggregate (*10 mm downsized aggregates) | Pycnometer method as per IS 2386, Part 3 |
| 4 | Silt and clay | Fine aggregates | As per IS 2386, Part 3 |
| 5 | Water absorption | Fine aggregates and coarse aggregate | Water absorption method as per IS 2386, Part 3 |
| 6 | Moisture | Fine aggregates and coarse aggregate | As per IS 2386, Part 3 |
| 7 | Choride test | Fine aggregates | Chloride test as per IS 3025, Part 32 |
| 8 | Aggregate impact | Coarse aggregates | As per IS 2386, Part 4 |
| 9 | Aggregate crushing | Coarse aggregates | As per IS 2386, Part 4 |
| 10 | Fineness modulus by sieve analysis | Fine aggregates and coarse aggregate | As per IS 2386, Part 1 [5], IS 383 [6], IS 460, Part 1 |
| 11 | Loose bulk density, compacted bulk density, voids | Fine aggregates and coarse aggregate | As per IS 2386, Part 1 |
| 12 | Scanning electron microscopy | Fine aggregates | EVO MA18 with Oxford EDS(X-act) |
| 13 | X-Ray Diffraction (XRD) anaysis | Fine aggregates | Rigaku Miniflex 600 (5th gen) |
| 14 | Soundness test | OPC, FA, GGBS | IS 12269 |
| 15 | Consistency, initial and final setting time | OPC, FA, GGBS | IS 12269 , IS 4031, Part 3 , IS 4031, Part 5 |
| 16 | Ultrasonic pulse velocity | Triple mix mortar cubes | Tico Ultrasonic Instrument |
| 17 | Compressive strength | Triple mix mortar cubes | IS 516, Part 1 |
| 18 | Mix design of triple mix SCC | Sequential trials | IS 10262, EFNARC 2005 |
| 19 | Slump flow, V funnel, T500 | Triple mix SCC | EFNARC 2005 |
| 20 | Compressive strength | 100 mm triple mix SCC cubes | IS 516, Part 1 |
| 21 | Splitting tensile strength | 150X300 mm triple mix SCC cylinders | IS 5816 |
| 22 | Flexural strength | 100X100X500 mm triple mix SCC beams | IS 516, Part 1 |
| 23 | Ultrasonic pulse velocity | 100 mm triple mix SCC cubes | IS 516, Part 5, Non-destructive testing of concrete, Tico Ultrasonic Instrument |
| 24 | Water absorption | 100 mm Triple mix SCC cubes | ASTM C642-21 |
| 25 | Wet density | Fresh triple mix SCC | Density cylinder |
| 26 | Dry density | 100 mm Triple mix SCC cubes | Dry weight and volume of the cube |
| 27 | Sorptivity | Triple mix SCC cylinders with 100 mm diameter and 50 mm height | ASTM C1585-20 |
| 28 | Sulphuric acid resistance | 100 mm Triple mix SCC cubes | ASTM C1898-20 |
| 29 | Sulphate attack test | 100 mm Triple mix SCC cubes | ASTM C267-20 |
| 30 | Rapid chloride permeability test | Triple mix SCC cylinders with 100 mm diameter and 50 mm height | ASTM C1202-22 |
| 31 | Scanning electron microscopy with energy dispersive X-ray spectroscopy | Triple mix SCC | EVO MA18 with Oxford EDS(X-act) |
| 32 | X-Ray Diffraction (XRD) anaysis | Triple mix SCC | Rigaku Miniflex 600 (5th gen) |
| 33 | pH | Water | pH meter |

Table 2: Tests for aggregates

| Sl. No. | Name of the test | Unit | Relevant code | Fine aggregates | | | Coarse aggregate (10 mm down) |
|---|---|---|---|---|---|---|---|
| | | | | River sand (zone 1) | Manufactured sand (zone 3) | Sea sand (zone 3) | |
| 1 | Fineness modulus by sieve analysis | - | IS: 2386 (Part 1), IS: 383, IS: 460 | 2.95 | 1.99 | 2.03 | 6.71 |
| 2 | a) Loose bulk density | kg/$m^3$ | IS: 2386 (Part 1) | 1420 | 1560 | 1470 | 1270 |
| | b) Compacted bulk density | kg/$m^3$ | IS: 2386 (Part 1) | 1600 | 1690 | 1570 | 1670 |
| 3 | Voids | % | IS: 2386 (Part 1) | 47 | 39 | 43.98 | 52 |
| 4 | Specific gravity | - | IS: 2386 (Part III) | 2.68 | 2.60 | 2.63 | 2.64 |
| 5 | Percentage of silt and clay by weight of the sand | % | IS:2386 (Part III) | 0.02 | 0.04 | 0 | - |

Table 3: Distinctive characteristics of the aggregates

| Sl. No. | Name of the test | Unit | Relevant code | Fine aggregates | | | Coarse aggregate (10 mm down) |
|---|---|---|---|---|---|---|---|
| | | | | River sand (zone 1) | Manufactured sand (zone 3) | Sea sand (zone 3) | |
| 1 | Percentage of silt and clay by volume of the sand | % | IS:2386 (Part III) | 2 | 5.4 | 0.37 | - |
| 2 | Water absorption | % | IS:2386 (Part III) | 0.6 | 1.01 | 1.21 | 0.75 |
| 3 | Moisture | % | IS:2386 (Part III) | 0 | 0 | 0 | 0.05 |
| 4 | Aggregate impact value | % | IS:2386 (Part IV) | - | - | - | 31.4 |
| 5 | Aggregate crushing value | % | IS:2386 (Part IV) | - | - | - | 33 |
| 6 | Chloride | % | - | 0.021 | 0.019 | 0.025 | - |
